# Impact of Alcohol-Induced Facial Flushing Phenotype on Alcohol Consumption Among Korean Adults: 2-Year Cross-Sectional Study

**DOI:** 10.2196/49826

**Published:** 2024-07-31

**Authors:** Bossng Kang, Changsun Kim, Seon-Hi Shin, Hyungoo Shin, Yongil Cho

**Affiliations:** 1 Department of Emergency Medicine, Hanyang University College of Medicine Seoul Republic of Korea; 2 Biostatistical Consulting and Research Lab Medical Research Collaborating Center Hanyang University Seoul Republic of Korea

**Keywords:** facial flushing, alcohol consumption, drinking behavior, alcohol, acetaldehyde, aldehyde dehydrogenase 2 polymorphism, East Asian

## Abstract

**Background:**

The alcohol-induced facial flushing phenotype (flushing) is common among East Asians. Despite a small intake of alcohol, they experience heightened levels of acetaldehyde, a group-1 carcinogen, which, in turn, causes unpleasant symptoms such as redness, acting as a robust protective mechanism against consuming alcohol. However, some individuals with this genetic trait exhibit weakened alcohol restraint, which increases the risk of developing alcohol-related cancers, such as esophageal and head or neck cancer, by several times. Although this flushing phenomenon is crucial for public health, there is a paucity of studies that have comprehensively investigated the effect of flushing or its genotype on alcohol consumption in a large group of East Asians while controlling for various sociodemographic and health-related variables at a country level.

**Objective:**

This 2-year cross-sectional study aims to explore the effect of flushing on drinking behavior in Koreans and to examine whether the effect varies across sociodemographic and health-related factors.

**Methods:**

We used data from the Korea National Health and Nutrition Examination Survey (KNHANES) for 2019 and 2020 conducted by the Korea Disease Control and Prevention Agency. Our sample comprised 10,660 Korean adults. The study investigated the association of 26 variables, including flushing, with drinking frequency and amount. The effect of flushing was examined with and without adjusting for the other 25 variables using multinomial logistic regression analysis. In addition, we tested the interaction effect with flushing and conducted a simple effect analysis. We used complex sample design elements, including strata, clusters, and weights, to obtain unbiased results for the Rao-Scott *χ*^2^ test, 2-tailed *t* test, and multinomial logistic regression analysis.

**Results:**

The suppressive effect of flushing was significant (*P*<.001) across all pronounced categories of alcohol consumption in 2019. The ranges of standardized regression slopes and odds ratios (ORs) were –6.70≥β≥–11.25 and 0.78≥OR≥0.50 for frequency and –5.37≥β≥–17.64 and 0.73≥OR≥0.36 for amount, respectively. The effect became somewhat stronger when adjusted for confounders. The effect also exhibited an overall stronger trend as the severity of alcohol consumption increased. The β values and ORs were consistently smaller in 2020 compared to the previous year. A simple effect analysis revealed a diminished alcohol-suppressive effect of flushing on alcohol consumption for specific groups (eg, those with low levels of education, limited family support, physical labor, or health-related issues).

**Conclusions:**

Our findings suggest that flushing suppresses drinking in Koreans overall but has little or no effect in certain susceptible populations. Therefore, health authorities should conduct targeted epidemiological studies to assess drinking patterns and disease profiles, particularly regarding alcohol-related cancers, and establish effective preventive measures tailored to this population.

## Introduction

### Background

The alcohol-induced facial flushing phenotype (hereafter flushing), characterized by facial redness even after a small intake of alcohol, is a prevalent genetic trait among East Asians. This distinctive response is attributed to a reduced metabolic capacity of the aldehyde dehydrogenase 2 (ALDH2) variant, which leads to elevated levels of acetaldehyde—a group-1 carcinogen [1-5]. Such a genetic variation is rare in individuals of European or African origin [2,3]. In addition to facial redness, individuals with this trait typically experience other unpleasant symptoms, including tachycardia, headache, and nausea, which collectively serve as a robust biological defense mechanism deterring alcohol intake [3,6-8]. However, recent limited observations indicate that some individuals with this genetic trait exhibit weakened alcohol restraint [1,9,10].

### This Study

Prolonged alcohol consumption among individuals with the ALDH2 variant significantly amplifies the risk of developing alcohol-related cancers, such as esophageal and head or neck cancers, by several times compared to those with active ALDH2 [11-14]. Therefore, it is imperative to identify the characteristics of individuals who exhibit flushing but have reduced drinking inhibitions, as they are particularly vulnerable to the detrimental effects of alcohol consumption due to heightened exposure to group-1 carcinogens [3-6]. However, to the best of our knowledge, comprehensive research investigating the impact of the flushing trait or its genotype on alcohol consumption behavior while accounting for various sociodemographic and health-related factors has not yet been conducted, particularly within a large East Asian population.

Hence, we hypothesized that flushing exerts a stronger influence on drinking behavior among East Asian individuals compared to other factors, and specific vulnerable subgroups may exhibit a mitigated effect. To test this hypothesis, we investigated the impact of flushing on alcohol consumption among a representative sample of Korean adults, drawn from the entire population. In addition, we explored the potential mediating influence of socioeconomic and health-related variables.

## Methods

### Study Design and Population

This 2-year cross-sectional study analyzed data from the annual Korea National Health and Nutrition Examination Survey (KNHANES) for 2019 and 2020 administered by the Korea Disease Control and Prevention Agency. Using a 2-stage stratified cluster sampling method, the survey sample was selected based on geographic region and housing type. Approximately 8000 individuals are recruited for the KNHANES each year. Although the KNHANES has been conducted since 1998, the health survey on flushing was only added in 2019. Thus, the data from the 2019 and 2020 surveys were included in this study. To address the potential impacts of the COVID-19 pandemic, the 2-year merged data set was analyzed to assess the interaction effect between year and flushing. Subsequently, unadjusted and adjusted logistic models were separately applied to the data for each individual year.

People aged ≤18 years and lifetime abstainers were excluded because they were children or did not know whether they experienced facial redness after drinking alcohol.

### Assessment of Variables

A total of 28 variables, including 25 sociodemographic and health-related variables, flushing, and 2 drinking behavior variables (frequency and amount of drinking), were used for the study. The drinking behavior data were obtained from the following survey questions: (1) How often did you drink over the past year (answered in 6 categories: did not drink in the past year, less than once a month, once a month, 2-4 times per month, 2-3 times per week, and ≥4 times per week)? (2) How many glasses did you drink when you drank over the past year (did not drink in the past year, 1-2 glasses, 3-4 glasses, 5-6 glasses, 7-9 glasses, and ≥10 glasses)? One glass is defined as 50 ml of soju (Korean traditional liquor, 20% alcohol by volume) or 220 ml of beer, the volume of the most common Korean beer glass size (5% alcohol by volume).

Flushing was determined by the following questions developed by Yokoyama et al [12,15]: (1) Do you currently have a constitution of your face turning red quickly after drinking as little as one glass of beer (soju; never, sometimes, often, or always)? The individuals who answered “always,” “often,” or “sometimes” to question (1) were defined as currently exhibiting flushing. Next, question (2) was asked to those who responded “never” or “not applicable” (did not drink within the past year) to question (1): (2) Did you experience your face turning red quickly after drinking as little as one glass of beer (or soju) during the 1 to 2 years after you started drinking (yes or no)? The individuals who responded “yes” to question (2) were regarded as formerly having exhibited flushing. These current and former individuals with flushing experience were assigned to the flushing group.

The survey data on the other 25 variables, selected from a literature review regarding the participants’ sociodemographic factors and health status, were obtained through in-person interviews or self-administration [16-20]. Age, BMI, and sleep time were continuous variables, while the rest were discrete variables. For some discrete variables, no responses were classified into a category.

### Statistical Analysis

#### Frequency and Mean Analyses

To comprehensively explore the baseline characteristics of the study sample, mean and frequency analyses were conducted. Differences between the 2 study years were examined using the Rao-Scott *χ*^2^ test and 2-tailed *t* test for complex survey data. In addition, a frequency analysis, accounting for strata, cluster, and weight, was performed to estimate the nationwide proportions of individuals exhibiting flushing among adults who had consumed alcohol.

#### Multinomial Logistic Regression Analysis

A 2-phase multinomial logistic regression analysis was performed to investigate potential variations in the impact of flushing on alcohol consumption behavior across different years. This analysis considered the influence of the COVID-19 pandemic and associated social distancing regulations. The model with drinking frequency or amount as the outcome variable included flushing, year, and their interaction to assess variations across years. Subsequently, multinomial logistic regression models were applied to each year’s data when the interaction effect was or was close to significant (*P*<.05). For each year, 2 models were formulated for each outcome variable: a simple model including only flushing as a predictor and a complex model incorporating additional sociodemographic and health-related factors.

#### Joint Analysis of Interaction and Simple Effects

Furthermore, interaction effects with flushing were tested for significant predictor variables in the complex models to investigate whether the effect of flushing on drinking behavior remained consistent across different levels of other predictor variables. When the interaction effect was significant at *P*<.05, a simple effect analysis was performed to examine the impact of flushing at each level of the other variable, separately. Predicted individual probabilities were computed for continuous variables with an interaction effect of *P*<.05.

#### Statistical Methods and Software

All statistical analyses were performed using SAS (version 9.4 TS Level 1M7; SAS Institute Inc). Given the limitations of the proportional odds assumption with numerous predictors, a more comprehensive multinomial logistic regression model was used [21]. Standardized regression coefficients (βs), odds ratios (ORs), and 95% CIs for predictor variables were presented to enhance direct comparability among the 26 predictor variables. Following the recommendation of KNHANES, complex sample design elements, including strata, clusters, and weights, were used, ensuring nationally representative population-based results for all inferential statistics generated in this study. Because the 2019 and 2020 data sets were combined within the same eighth survey cycle, a new weight was calculated for analysis by multiplying 0.5 with the basic association weight vaxriable, *wt_itvex*, for each year’s data set. Furthermore, to prevent any bias in SEs that may arise when analyzing only the data of the target group after excluding the rest of the data set, a group variable was created to distinguish the individuals under investigation from those aged ≤18 years and lifetime abstainers. This variable was specified in the SAS statements for frequency and mean analyses, as well as for multinomial logistic regression analysis.

### Ethical Considerations

The original data collection was approved by the institutional review board of the Korea Disease Control and Prevention Agency, and this study received exemption from review by Hanyang University Guri Hospital’s institutional review board.

## Results

### Baseline Characteristics

A total of 15,469 individuals participated in the KNHANES 2019 and 2020 surveys. Of these, 10,660 (68.91%) individuals were included in the analysis, excluding children (aged ≤18 years) and lifetime abstainers. Overall, 5506 cases from 2019 and 5154 cases from 2020 were used in the complex multinomial logistic regression models. The baseline characteristics of participants are shown in Table 1. In 2019, the number of people categorized into the flushing group was 2489 (45.25%), while in 2020, it was 2083 (40.46%). The corresponding population proportions were estimated to be 44.42% (8,589,410/19,337,305) for 2019 and 40.11% (7,867,415/19,612,917) for 2020, respectively.

**Table 1:** Baseline characteristics of the study sample and population: Korean adults from 2019-2020 Korea National Health and Nutrition Examination Survey (KNHANES)^a^.

Variable			2019				2020				*P* value
			Value		Participants, n		Value		Participants, n		
Age (y), mean (SD)			50.12 (16.5)		5506		50.49 (16.9)		5154		.68
BMI, mean (SD)			23.93 (3.6)		5485		24.21 (3.8)		5094		<.001
Sleeping hours per day on days off, mean (SD)			7.40 (1.7)		5502		7.44 (1.7)		5149		.47
**Sex, n (%)**					5506				5154		.39
	Male	2621 (47.6)				2522 (48.9)					
	Female	2885 (52.4)				2632 (51.1)					
**Spouse, n (%)**					5506				5154		.09
	Never married	1028 (18.7)				1119 (21.7)					
	Living with a spouse	3819 (69.4)				3360 (65.2)					
	Living without a spouse	659 (12)				675 (13.1)					
**Education, n (%)**					5506				5154		.03
	≤Elementary school diploma	789 (14.3)				665 (12.9)					
	Middle-school diploma	477 (8.7)				433 (8.4)					
	High-school diploma	1830 (33.2)				1758 (34.1)					
	≥University diploma	2186 (39.7)				1948 (37.8)					
	Others, including no response	224 (4.1)				350 (6.8)					
**Family size (number of members), n (%)**					5506				5154		.18
	1	666 (12.1)				646 (12.5)					
	2	1736 (31.5)				1529 (29.7)					
	3	1417 (25.7)				1325 (25.7)					
	4	1292 (23.5)				1198 (23.2)					
	5	322 (5.8)				345 (6.7)					
	≥6	73 (1.3)				111 (2.2)					
**Area type, n (%)**					5506				5154		.71
	City district	4465 (81.1)				4178 (81.1)					
	Town or village	1041 (18.9)				976 (18.9)					
**Occupation, n (%)**					5506				5154		.23
	Administrator or professional	786 (14.3)				724 (14.1)					
	Office worker	646 (11.7)				545 (10.6)					
	Service or sales worker	680 (12.4)				684 (13.3)					
	Farmer or fisherman	153 (2.8)				159 (3.1)					
	Mechanic or technician	586 (10.6)				474 (9.2)					
	Simple labor worker	466 (8.5)				404 (7.8)					
	Unemployed (housewife, student)	1943 (35.3)				1802 (35)					
	Other, including no response	246 (4.5)				362 (7)					
**Household income, n (%)**					5486				5140		.95
	Low	721 (13.1)				624 (12.1)					
	Low-middle	953 (17.4)				868 (16.9)					
	Middle	1124 (20.5)				1096 (21.3)					
	Middle-high	1312 (23.9)				1242 (24.2)					
	High	1376 (25.1)				1310 (25.5)					
**Houses owned, n (%)**					5505				5153		.18
	0	1950 (35.4)				1661 (32.2)					
	1	2918 (53)				2783 (54)					
	≥2	637 (11.6)				709 (13.8)					
**Type of health insurance, n (%)**					5506				5154		.06
	Residence-based national health insurance	1503 (27.3)				1549 (30.1)					
	Work-based national health insurance	3813 (69.3)				3392 (65.8)					
	Medical care	190 (3.5)				213 (4.1)					
**Private health insurance, n (%)**					5480				5120		.59
	Have	4544 (82.9)				4272 (83.4)					
	Do not have	936 (17.1)				848 (16.6)					
**Smoking, n (%)**					5502				5150		.75
	Daily	896 (16.3)				820 (15.9)					
	Occasionally	154 (2.8)				139 (2.7)					
	Smoked before but not presently	1383 (25.1)				1317 (25.6)					
	Never	3069 (55.8)				2874 (55.8)					
**Weight control effort within the past 1 year, n (%)**					5505				5153		.01
	To lose weight	2283 (41.5)				2148 (41.7)					
	To maintain weight	1096 (19.9)				1108 (21.5)					
	To gain weight	302 (5.5)				226 (4.4)					
	Never tried	1824 (33.1)				1671 (32.4)					
**Limitation on life activities due to poor health or disability, n (%)**					5506				5154		<.01
	Have	358 (6.5)				321 (6.2)					
	Do not have	4928 (89.5)				4492 (87.2)					
	Others, including no response	220 (4)				341 (6.6)					
**Feeling stressed, n (%)**					5502				5146		.59
	Very frequently	257 (4.7)				264 (5.1)					
	Frequently	1236 (22.5)				1187 (23.1)					
	Occasionally	3204 (58.2)				2956 (57.4)					
	Seldomly	805 (14.6)				739 (14.4)					
**Suicide attempt within the past 1 year, n (%)**					5503				5149		.96
	Yes	20 (0.4)				25 (0.5)					
	No	5483 (99.6)				5124 (99.5)					
**Psychiatric consultation within the past 1 year, n (%)**					5503				5149		.81
	Yes	175 (3.2)				177 (3.4)					
	No	5328 (96.8)				4972 (96.6)					
**Number of major fatal illnesses^b^, n (%)**					5506				5154		.45
	1	464 (8.4)				428 (8.3)					
	≥2	51 (0.9)				38 (0.7)					
	Others, including no response	4991 (90.6)				4688 (91)					
**Osteoarthritis and rheumatoid arthritis diagnosis, n (%)**					5506				5154		.24
	Present	582 (10.6)				524 (10.2)					
	Others, including no response	4924 (89.4)				4630 (89.8)					
**Hypertension diagnosis, n (%)**					5506				5154		.54
	Present	1267 (23)				1242 (24.1)					
	Others, including no response	4239 (77)				3912 (75.9)					
**Dyslipidemia diagnosis, n (%)**					5506				5154		.03
	Present	989 (18)				1082 (21)					
	Others, including no response	4517 (82)				4072 (79)					
**Osteoporosis diagnosis, n (%)**					5506				5154		.42
	Present	327 (5.9)				316 (6.1)					
	Others, including no response	5179 (94.1)				4838 (93.9)					
**Diabetes diagnosis, n (%)**					5506				5154		.16
	Present	496 (9)				530 (10.3)					
	Others, including no response	5010 (91)				4624 (89.7)					
**Drinking frequency within the past 1 year, n (%)**					5503				5153		.08
	Never	1070 (19.4)				1103 (21.4)					
	<Once per month	1106 (20.1)				1076 (20.9)					
	Once per month	690 (12.5)				581 (11.3)					
	2-4 times per month	1330 (24.2)				1215 (23.6)					
	2-3 times per week	897 (16.3)				847 (16.4)					
	≥4 times per week	410 (7.5)				331 (6.4)					
**Drinking amount within the past 1 year (number of glasses), n (%)**					5503				5153		.17
	0	1070 (19.4)				1103 (21.4)					
	1-2	1570 (28.5)				1469 (28.5)					
	3-4	940 (17.1)				822 (16)					
	5-6	629 (11.4)				507 (9.8)					
	7-9	686 (12.5)				655 (12.7)					
	≥10	608 (11)				597 (11.6)					
**Alcohol-induced facial flushing^c^, n (%)**											<.001
	Present	2489 (45.2)		5501		2083 (40.5)		5148			
	Absent	3012 (54.8)		5501		3065 (59.5)		5148			
	Present^d^	8,589,410 (44.4)		19,337,305		7,867,415 (40.1)		19,612,917			
	Absent^d^	10,747,895 (55.6)		19,337,305		11,745,502 (59.9)		19,612,917			

^a^The sample for the KNHANES in 2019 and 2020 was selected through a complex sample design involving a multistage stratified cluster probability sampling method each year. The target population for the study was individuals aged ≥19 years and nonlifetime abstainers from alcohol consumption. *P* values for discrete variables were obtained from the Rao-Scott *χ*^2^ test, while those for continuous variables were obtained from the *t* test. To ensure unbiased results, complex sample design elements, including strata, clusters, and weights, were incorporated in both the Rao-Scott *χ*^2^ test and *t* test.

^b^The number of diagnoses by physicians for stomach, liver, colon, breast, cervical, lung, thyroid, and other cancers; stroke; and myocardial infarction or angina.

^c^A genetic predisposition linked to aldehyde dehydrogenase 2 deficiency, manifesting as facial redness even with small amounts of alcohol. The identification of this phenotype relies on a 2-step questionnaire outlined in the *Methods* section.

^d^The weighted statistics obtained to estimate population parameters.

### Impact of Flushing on Alcohol Consumption Behavior by Year

The results of the multinomial logistic regression analysis using the 2-year merged data set revealed significant or close-to-significant interaction effects between flushing and year on drinking behavior. The test statistics and *P* values were as follows: *F*_5,341_=3.02, *P*=.01 for drinking frequency and *F*_5,341_=2.01, *P*=.08 for drinking amount. Tables S1 and S2 in Multimedia Appendix 1 provide further details. It is worth noting that the COVID-19 pandemic and strict social distancing regulations were prevalent in 2020. It is likely that the effects of the 25 confounders on alcohol consumption behavior, as well as their relationships with flushing, varied across the 2 years due to extreme social changes. Therefore, further analyses were conducted separately for each year.

### Impact of Flushing on Alcohol Consumption Behavior Among Koreans

#### Frequency of Alcohol Consumption

The effects of the simple and complex multinomial logistic regression models with drinking frequency as the dependent variable are presented in Table 2. The presence of flushing showed a significantly negative correlation with the frequency of alcohol consumption at all contrasts (all ORs were <1, and CIs did not include the value of 1), except for the lowest category of “<1 per month” versus “did not drink within the past year.” For instance, the odds of “drinking 2-3 times per week” versus “did not drink in the past year” were 0.56 times lower in the flushing group than in the nonflushing group (OR 0.56, 95% CI 0.45-0.69). The odds further decreased to 0.47 times lower (95% CI 0.37-0.59) when other confounding variables were taken into account in the complex model. The ORs tended to decrease overall as the drinking frequency increased, and they became smaller in the same contrasts in 2020, when the COVID-19 pandemic and social distancing regulations prevailed (Table 2).

**Table 2:** Effect of alcohol-induced facial flushing^a^ on drinking frequency among Korean adults from 2019-2020 Korea National Health and Nutrition Examination Survey (KNHANES)^b^.

Predictor variable and category vs reference category			Drinking frequency (reference=did not drink in the past year)																							
			<Once per month					Once per month					2-4 times per month					2-3 times per week					≥4 times per week			
			β^c^; OR^d^ (95% CI)		*P* value		β; OR (95% CI)			*P* value		β; OR (95% CI)			*P* value		β; OR (95% CI)			*P* value		β; OR (95% CI)			*P* value	
**Alcohol-induced facial flushing (2019)**																										
	Presence vs absence of simple model	0.77; 1.07 (0.85-1.34)		.57		–2.97; 0.78 (0.62-0.98)			.03		–6.70; 0.56 (0.47-0.68)			<.001		–6.88; 0.56 (0.45-0.69)			<.001		–8.12; 0.50 (0.39-0.64)			<.001		
	Presence vs absence of complex model	1.09; 1.10 (0.87-1.39)		.43		–3.06; 0.77 (0.61-0.98)			.03		–7.32; 0.54 (0.44-0.65)			<.001		–8.83; 0.47 (0.37-0.59)			<.001		–11.25; 0.38 (0.29-0.51)			<.001		
**Alcohol-induced facial flushing (2020)**																										
	Presence vs absence of simple model	–1.46; 0.88 (0.73-1.07)		.20		–5.93; 0.60 (0.47-0.76)			<.001		–9.80; 0.43 (0.36-0.52)			<.001		–13.56; 0.31 (0.25-0.39)			<.001		–10.23; 0.41 (0.30-0.56)			<.001		
	Presence vs absence of complex model	–1.62; 0.87 (0.70-1.08)		.21		–7.07; 0.54 (0.42-0.70)			<.001		–12.02; 0.35 (0.28-0.44)			<.001		–17.22; 0.23 (0.17-0.29)			<.001		–15.54; 0.26 (0.18-0.37)			<.001		

^a^A genetic predisposition linked to aldehyde dehydrogenase 2 deficiency, manifesting as facial redness even with small amounts of alcohol. The identification of this phenotype relies on a 2-step questionnaire outlined in the *Methods* section.

^b^The sample for the KNHANES in 2019 and 2020 was selected through a complex sample design involving a multistage stratified cluster probability sampling method each year. The target population for the study was individuals aged ≥19 years and non–lifetime abstainers from alcohol consumption. Multinomial logistic regression analysis was conducted, incorporating complex sample design elements, including strata, clusters, and weights, to ensure unbiased results. The *P* values were obtained for the unstandardized regression slope coefficients, whose corresponding odds ratios are presented in the table.

^c^The statistic β represents a standardized regression coefficient, the absolute value of which reflects the degree of association between the predictor variable and the outcome variable (drinking frequency), which enhances direct comparability among multiple predictor variables in the multinomial logistic regression analysis.

^d^OR: odds ratio.

#### Amount of Alcohol Consumed

The results from the simple and complex multinomial logistic regression models with drinking amount as their dependent variable are presented in Table 3. The effect of flushing on the amount of drinking showed a similar but slightly stronger pattern (all slopes were significantly negative, ORs were <1, and CIs did not include the value of 1, except for the lowest category of “drinking 1-2 glasses each time” vs “did not drink within the past year” in 2019). The OR decreased consistently as the drinking amount increased (Table 3).

When sociodemographic and health-related variables were included in the model as explanatory variables in addition to flushing (complex model), the OR decreased. The ORs were smaller in the same contrasts in 2020 compared to 2019 (Table 3).

**Table 3:** Effect of alcohol-induced facial flushing^a^ on drinking amount among Korean adults from 2019-2020 Korea National Health and Nutrition Examination Survey (KNHANES)^b^.

Predictor variable and category vs reference category			Drinking amount (glasses per drinking occasion; reference=did not drink in the past year)																							
			1-2					3-4					5-6					7-9					≥10			
			β^c^; OR^d^ (95% CI)		*P* value		β; OR (95% CI)			*P* value		β; OR (95% CI)			*P* value		β; OR (95% CI)			*P* value		β; OR (95% CI)			*P* value	
**Alcohol-induced facial flushing (2019)**																										
	Presence vs absence of simple model	0.44; 1.04 (0.86-1.26)		.70		–3.61; 0.73 (0.60-0.89)			.002		–5.37; 0.63 (0.50-0.79)			<.001		–7.54; 0.52 (0.40-0.69)			<.001		–11.84; 0.36 (0.28-0.47)			<.001		
	Presence vs absence of complex model	1.25; 1.11 (0.91-1.36)		.29		–5.33; 0.63 (0.52-0.78)			<.001		–8.33; 0.49 (0.38-0.63)			<.001		–12.01; 0.36 (0.26-0.49)			<.001		–17.64; 0.22 (0.16-0.30)			<.001		
**Alcohol-induced facial flushing (2020)**																										
	Presence vs absence of simple model	–3.11; 0.77 (0.64-0.92)		.003		–7.89; 0.51 (0.41-0.62)			<.001		–8.73; 0.47 (0.37-0.60)			<.001		–12.01; 0.36 (0.28-0.45)			<.001		–12.42; 0.34 (0.27-0.44)			<.001		
	Presence vs absence of complex model	–3.10; 0.77 (0.63-0.94)		.009		–10.24; 0.41 (0.33-0.52)			<.001		–12.36; 0.34 (0.26-0.46)			<.001		–17.26; 0.23 (0.17-0.30)			<.001		–18.40; 0.20 (0.15-0.28)			<.001		

^a^A genetic predisposition linked to aldehyde dehydrogenase 2 deficiency, manifesting as facial redness even with small amounts of alcohol. The identification of this phenotype relies on a two-step questionnaire outlined in the *Methods* section.

^b^The sample for the KNHANES in 2019 and 2020 was selected through a complex sample design involving a multistage stratified cluster probability sampling method each year. The target population for the study was individuals aged ≥19 years and non–lifetime abstainers from alcohol consumption. Multinomial logistic regression analysis was conducted, incorporating complex sample design elements, including strata, clusters, and weights, to ensure unbiased results.

^c^The statistic β represents a standardized regression coefficient, the absolute value of which reflects the degree of association between the predictor variable and the outcome variable (drinking amount), which enhances direct comparability among multiple predictor variables in the multinomial logistic regression analysis. The *P* values were obtained for the unstandardized regression slope coefficients, whose corresponding odds ratios are presented in the table.

^d^OR: odds ratio.

### Sociodemographic and Health-Related Variables Affecting Alcohol Consumption Behavior

#### Frequency of Alcohol Consumption

In the complex model, 5 sociodemographic and 7 health-related characteristics besides flushing were significantly associated with drinking frequency in 2019 (Table S3 in Multimedia Appendix 1). Age, education level, limitation on life activities, and the number of major fatal illnesses showed a negative association in ≥2 contrasts, albeit to varying degrees, whereas smoking, feeling stressed, and hypertension diagnosis were positively associated with drinking frequency. Male individuals tended to drink more frequently than female individuals. Individuals covered by work-based national health insurance drank more frequently than medical-care beneficiaries, as observed in the contrast of less than once per month. People living without a spouse consumed alcohol less frequently than those who were never married in the contrast of once per month. BMI exhibited a significantly negative association with drinking frequency in a single contrast of once per month. There were no statistically significant contrasts in occupation, although the overall test for this variable was significant according to the type 3 analysis of effects (Table S3 in Multimedia Appendix 1).

Meanwhile, in the complex model using the 2020 data set, the number of major fatal illnesses was no longer statistically significant. Instead, family size, household income, number of houses owned, private health insurance, and weight control effort were newly identified as significant predictor variables. Family size was negatively associated with drinking frequency, whereas household income, number of houses owned, and private health insurance were positively associated. They were all statistically significant in ≥2 contrasts. In addition, individuals making efforts to lose weight drank more frequently than those who never tried weight control efforts in the single contrast of “2-4 times per month” (Table S4 in Multimedia Appendix 1).

#### Amount of Alcohol Consumed

The complex model, using drinking amount as the outcome variable with the 2019 data set, yielded results generally comparable to those obtained from its counterpart using drinking frequency with the same data set. In total, 13 variables showed statistical significance, except for flushing. Out of the 12 confounding variables initially identified as significant in the model with drinking frequency, 3 (25%) variables—spouse, type of health insurance, and feeling stressed—were no longer significant. In addition, 4 new variables—private health insurance, weight control efforts, suicide attempt, and off-day sleep hours—emerged as significant predictor variables (Table S5 in Multimedia Appendix 1).

Age, education level, and limitations in life activities exhibited negative associations across multiple contrasts in varying degrees, while smoking, private health insurance, and hypertension diagnosis were positively associated with alcohol consumption. Male individuals tended to consume more alcohol than their counterparts. Individuals making efforts to gain weight drank less than those who never attempted weight control efforts in the 2 contrasts of consuming 3 to 4 glasses or 7 to 9 glasses per drinking occasion. Office workers and mechanics or technicians tended to consume more alcohol, while unemployed individuals such as housewives or students consumed less alcohol compared to administrators or professionals.

Regarding the results based on the 2020 data set, 10 (77%) out of 13 variables identified as significant in the analysis of the 2019 data set remained significant. Three variables—weight control efforts, off-day sleep hours, and suicide attempt—were no longer significant. However, 3 new variables—household income, type of health insurance, and dyslipidemia diagnosis—emerged as significant confounding variables. Household income was positively associated in multiple contrasts, while dyslipidemia diagnosis was negatively associated in one contrast with alcohol consumption. Individuals covered by work-based national health insurance drank more than medical-care beneficiaries in the contrast of 7-9 glasses (Table S6 in Multimedia Appendix 1).

### Changes in the Effect of Flushing on Alcohol Consumption Behavior at Different Levels of Sociodemographic and Health-Related Variables

The interaction effects between flushing and other explanatory variables were investigated to determine whether the restraining effect of flushing on drinking behavior would vary across different levels of the sociodemographic and health-related characteristics. Only the variables whose associations with drinking behavior were significant were tested for this analysis. As a result, 5 variables exhibited a significant interaction effect with flushing in the analysis with drinking frequency as the outcome variable, while 1 variable showed significance in the analysis with drinking amount as the outcome variable, using the 2019 data set (Tables 4 and 5). The results from the 2020 data set are displayed in Tables S7 and S8 in Multimedia Appendix 1. Subsequently, a simple effect analysis was conducted to evaluate the impact of flushing on drinking behavior for each category of discrete variables with significant interaction.

For instance, referring to Table 4, the suppressive effect of flushing on drinking behavior significantly decreases as the level of education decreases, particularly evident in the 3 most pronounced contrasts in alcohol intake (2-4 times per month to ≥4 times per week vs the reference). The odds of consuming alcohol “2-4 times a month,” “2-3 times a week,” and “≥4 times a week” compared to “did not drink in the past year” were 0.51, 0.44, and 0.46 times lower for the flushing group than the nonflushing group, respectively, among those with a high-school diploma. However, these restraining effects completely disappeared in the flushing group with an elementary school diploma or a lower level of education (OR 1.33, 95% CI 0.84-2.09; OR 1.18, 95% CI 0.64-2.19; and OR 0.79, 95% CI 0.45-1.38). This pattern of attenuation was also observed for the drinking frequency of the 2020 data set (Table S7 in Multimedia Appendix 1). The suppressive effect of flushing on drinking amount noticeably diminished among individuals with a middle-school diploma or lower level of education (Table S8 in Multimedia Appendix 1). Similarly, as the level of household income decreased, the flushing effect tended to somewhat diminish (Table S7 in Multimedia Appendix 1).

Further, the flushing effect diminished among individuals who were not living with a spouse or never married compared to those living with a spouse, as seen in the 3 largest contrasts (Table 4). The flushing effect completely disappeared among individuals whose family size was ≥6 members, while it persisted in categories with fewer than 6 members (Table S7 in Multimedia Appendix 1). Among different occupational groups, the alcohol-restraining effect of flushing noticeably disappeared in farmers and fishermen in the 3 largest contrasts of drinking frequency, while the effect remained relatively stable among administrators or professionals (Table S7 in Multimedia Appendix 1). Male individuals exhibited a more pronounced suppressive effect of flushing compared to female individuals, as indicated by notably smaller ORs observed at the last 4 levels of drinking frequency in both years (Table 4 and Table S7 in Multimedia Appendix 1). This gender disparity in the impact of flushing on drinking frequency is further illustrated in Table S9 of Multimedia Appendix 1, which was based on a gender-stratified analysis conducted using the 2019 data set, presumed to be unaffected by the pandemic. Initially, a significant interaction effect between gender and flushing was observed solely during the examination of drinking frequency.

In terms of health-related issues, the alcohol-suppressing effect of flushing on drinking behavior appeared weaker among patients with hypertension compared to others (Table 4). The flushing effect completely disappeared among occasional smokers (Table 5 and Table S7 in Multimedia Appendix 1) or diminished among individuals without private health insurance, except for those with the most severe level of alcohol consumption (Table S8 in Multimedia Appendix 1).

**Figure 1:**
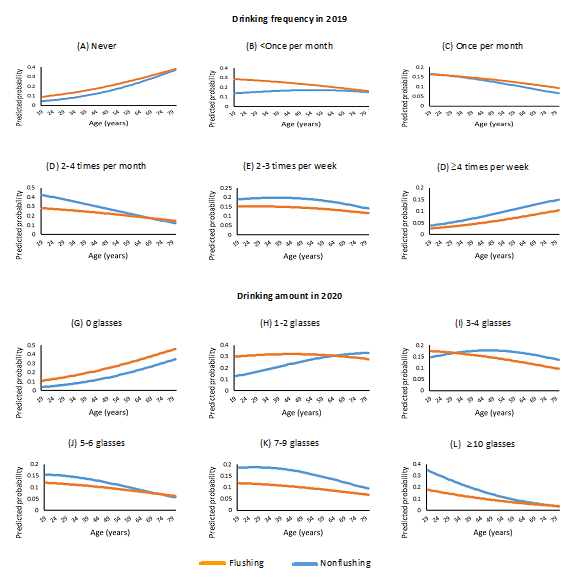
Changes in the effect of alcohol-induced facial flushing on alcohol consumption behavior with age among Korean adults in the 2019-2020 Korea National Health and Nutrition Examination Survey (KNHANES). The line graphs are presented exclusively for the results where the interaction effects of flushing and age were statistically significant at the .05 level, observed with the dependent variables of drinking frequency in 2019 and drinking amount in 2020, respectively. The expected probabilities of flushing versus nonflushing groups as a function of age at each level of drinking frequency (A-F) and drinking amount (G-L). (A and G) The expected probability of the flushing group is slightly higher than that of its counterpart, and the trend increases with age for both groups. (B and H) The flushing group shows a noticeably higher expected probability at younger ages compared to its counterpart, but the gap gradually decreases as age increases. (C) The difference between the 2 groups is minimal, and both exhibit decreasing trends with increasing age. (I) The flushing group exhibits a linear decrease in the expected probability, while its counterpart shows a negative quadratic trend with increasing age. (D), (E), (J), (K), and (L): in all these categories representing relatively high degrees of drinking frequency and amount, the expected probability of the flushing group is lower than that of its counterpart. This group difference appears larger at younger ages but, by and large, decreases as age increases. (F) The expected probabilities of both groups increase steadily as age increases at the highest level of drinking frequency. This suggests that the alcohol consumption behavior of those drinking as frequently as ≥4 times per week would be quite different from that of the rest, regardless of the presence of flushing.

Figure 1 shows how the flushing effect varied as a function of age.

**Table 4:** Changes in the impact of alcohol-induced facial flushing^a^ on drinking frequency at different levels of demographic and health-related variables among Korean adults from 2019 Korea National Health and Nutrition Examination Survey (KNHANES; simple effects analysis)^b^.

Predictor variable		Drinking frequency (reference=did not drink in the past year), OR^c^ (95% CI)							*F* test (*df*)			*P* value	
		<Once per month	Once per month	2-4 times per month	2-3 times per week		≥4 times per week						
Age^d^ × flushing		—^e^	—	—	—		—	3.12 (5,341)			.009		
**Sex × flushing**										2.86 (5,341)			.02
	Male	1.13 (0.77-1.64)	0.61 (0.43-0.87)	0.45 (0.33-0.60)		0.39 (0.28-0.53)	0.35 (0.25-0.50)						
	Female	1.04 (0.81-1.35)	0.85 (0.64-1.14)	0.58 (0.45-0.76)		0.70 (0.53-0.93)	0.67 (0.37-1.21)						
**Spouse × flushing**										2.29 (10,336)			.01
	Never married	1.18 (0.62-2.25)	0.59 (0.32-1.08)	0.43 (0.23-0.79)		0.65 (0.35-1.19)	0.53 (0.23-1.23)						
	Living with a spouse	0.92 (0.71-1.20)	0.84 (0.63-1.13)	0.61 (0.49-0.75)		0.48 (0.37-0.63)	0.46 (0.34-0.61)						
	Living without a spouse	1.63 (1.02-2.60)	0.77 (0.41-1.42)	0.58 (0.34-0.98)		1.00 (0.54-1.85)	0.79 (0.36-1.76)						
**Education × flushing**										1.75 (20,326)			.03
	≤Elementary school diploma	0.87 (0.57-1.32)	1.02 (0.52-2.01)	1.33 (0.84-2.09)		1.18 (0.64-2.19)	0.79 (0.45-1.38)						
	Middle-school diploma	1.14 (0.58-2.22)	0.76 (0.34-1.70)	0.40 (0.22-0.73)		0.67 (0.34-1.33)	0.43 (0.21-0.88)						
	High-school diploma	1.17 (0.80-1.71)	0.75 (0.52-1.07)	0.51 (0.37-0.71)		0.44 (0.30-0.64)	0.46 (0.29-0.72)						
	≥University diploma	0.99 (0.70-1.39)	0.67 (0.47-0.96)	0.53 (0.39-0.72)		0.55 (0.38-0.80)	0.42 (0.25-0.73)						
**Hypertension diagnosis × flushing**										2.25 (5,341)			.049
	Present	1.08 (0.70-1.67)	0.98 (0.56-1.70)	0.76 (0.53-1.09)		0.86 (0.59-1.25)	0.48 (0.30-0.76)						
	Others, including no response	1.03 (0.79-1.33)	0.72 (0.56-0.92)	0.51 (0.42-0.63)		0.49 (0.38-0.62)	0.51 (0.37-0.70)						

^a^A genetic predisposition linked to aldehyde dehydrogenase 2 deficiency, manifesting as facial redness even with small amounts of alcohol. The identification of this phenotype relies on a 2-step questionnaire outlined in the *Methods* section.

^b^The sample for the KNHANES in 2019 and 2020 was selected through a complex sample design involving a multistage stratified cluster probability sampling method each year. The target population for the study was individuals aged ≥19 years and non–lifetime abstainers from alcohol consumption in 2019. Multinomial logistic regression analysis was conducted, followed by a simple effect analysis for variables showing a significant interaction effect with flushing. To ensure unbiased results, complex sample design elements, including strata, clusters, and weights, were incorporated in the multinomial logistic regression analysis. The *F* value represents the test statistic for type 3 analysis of the interaction effect between the 2 variables under investigation. A significant interaction effect was observed for the variable “number of major fatal illnesses” but omitted due to unstable results caused by zero or near-zero observations in certain categories reflecting the rarity in the population.

^c^OR: odds ratio.

^d^Although age had a significant interaction with flushing, a simple effect analysis could not be performed because age was a continuous variable. Instead, the interaction effect between flushing and age could be visually observed by comparing the difference in expected probability between the flushing group and the nonflushing group over age at each level of drinking frequency (Figure 1).

^e^Not applicable because age was a continuous variable.

**Table 5:** Changes in the impact of alcohol-induced facial flushing^a^ on drinking amount at different levels of demographic and health-related variables among Korean adults from 2019 Korea National Health and Nutrition Examination Survey (KNHANES; simple effects analysis)^b^.

Predictor variable		Drinking amount (glasses per drinking occasion; reference=did not drink in the past year), OR^c^ (95% CI)					*F* test (*df*)		*P* value	
		1-2	3-4	5-6	7-9	≥10				
**Smoking × flushing**								2.20 (15,331)		.006
	Daily	2.57 (1.25-5.29)	0.74 (0.40-1.38)	0.33 (0.18-0.61)	0.37 (0.20-0.68)	0.20 (0.12-0.33)				
	Occasionally	0.95 (0.14-6.48)	0.67 (0.10-4.37)	1.36 (0.21-8.90)	0.68 (0.11,4.28)	0.32 (0.06-1.77)				
	Smoked before but not presently	1.12 (0.72-1.74)	0.67 (0.45-1.02)	0.43 (0.26-0.69)	0.42 (0.27-0.66)	0.31 (0.18-0.53)				
	Never	1.05 (0.83-1.32)	0.65 (0.50-0.85)	0.75 (0.53-1.06)	0.38 (0.25-0.58)	0.32 (0.20-0.51)				

^a^A genetic predisposition linked to aldehyde dehydrogenase 2 deficiency, manifesting as facial redness even with small amounts of alcohol. The identification of this phenotype relies on a 2-step questionnaire outlined in the *Methods* section.

^b^The sample for the KNHANES in 2019 and 2020 was selected through a complex sample design involving a multistage stratified cluster probability sampling method each year. The target population for the study was individuals aged ≥19 years and non–lifetime abstainers from alcohol consumption in 2019. Multinomial logistic regression analysis was conducted, followed by a simple effect analysis for the variable showing a significant interaction effect with flushing. To ensure unbiased results, complex sample design elements, including strata, clusters, and weights, were incorporated in the multinomial logistic regression analysis. The *F* value represents the test statistic for type 3 analysis of the interaction effect between the 2 variables under investigation. Significant interaction effects were observed for the variables “number of major fatal illnesses” and “suicide attempt” but omitted due to unstable results caused by zero or near-zero observations in certain categories reflecting the rarity in the population.

^c^OR: odds ratio.

## Discussion

### Principal Findings

Flushing was estimated to manifest in 40.11% or 44.42% of the Korean population aged ≥19 years who have consumed alcohol. People with this phenotype tended to consume significantly less alcohol in terms of drinking frequency and amount than people without it. The suppressive effect of flushing was significant in all categories of alcohol consumption except for the lowest one (<once per month or 1-2 glasses per occasion) in 2019 (0.78≥OR≥0.50 for frequency; 0.73≥OR≥0.36 for amount). This restraining tendency grew stronger as the drinking frequency and amount increased. When sociodemographic and health-related variables were added to the models, the restraining effect of flushing on drinking behavior remained statistically significant, and its effect size remained relatively large or even slightly larger (0.77≥OR≥0.38 for frequency; 0.63≥OR≥0.22 for amount). Put differently, with various contextual characteristics such as age, sex, economic status, education, family size, occupation, smoking, activity limitation, and various illnesses held constant, those with flushing had a significantly lower drinking frequency and amount than those without it. The effect also showed an overall stronger trend as the severity of alcohol consumption increased. Meanwhile, when comparing results year by year, the suppressive impact of flushing became more pronounced in 2020 compared to 2019; the ORs consistently showed a decrease in 2020 compared to the previous year. This suggests that the impact of the COVID-19 pandemic and associated social distancing measures on alcohol consumption suppression appeared to be relatively greater in the flushing group. All of these were illustrated by the magnitude of their standardized regression slopes and ORs; this phenotype exhibited one of the most substantial effect sizes, alongside age, sex, and smoking.

Although many studies have reported similar findings, our results are unique and important in the following aspects. First, the findings of this study may reflect the characteristics of East Asians in general because a sufficient and representative sample of the Korean population was used in the analysis. Prior studies focused on subgroups of the general population, such as college students, young adults, and middle-aged men, and were generally limited to <500 participants [7,22-25]. The studies conducted by Baik et al [26] and Au Yeung et al [8] examined >2800 and 4800 participants, respectively, but both targeted specific male age ranges, thus potentially limiting generalizability. Second, in addition to flushing, 25 sociodemographic and health-related factors known to possibly affect drinking behavior were included in the analysis, making it possible to interpret the flushing effect with sufficient adjustment for the effects of contextual factors. Previous studies involved a single variable (ALDH2 deficiency) or included several other predictors [7,8,22-26]. Third, the study measured the magnitude of the impact of flushing on alcohol consumption behavior by directly comparing it with those of other competing factors in terms of standardized regression coefficients.

Further, this study demonstrated that the magnitude of the flushing effect on alcohol drinking behavior could differ substantially and is contingent on the level of sociodemographic and health-related variables. This outcome can provide valuable information to public health policy makers. Initially, we observed a significant suppressing effect of flushing on alcohol consumption in simple and complex models. However, the subsequent joint analysis of interaction and simple effects revealed the attenuation of this effect in various categories, including adults with low education levels, adults with low levels of household income, adults living without a spouse, adults with ≥6 family members, farmers or fishermen, and adults with certain health-related issues (eg, hypertension diagnosis, absence of private insurance, and occasional smoking). This attenuation may be attributed to 2 factors. First, the flushing group’s alcohol intake increased, aligning it with a similar drinking level to the nonflushing group. Second, the nonflushing group consumed less alcohol due to the overall lower level of drinking in those specific categories. When examining the level of drinking in these categories as the strength associated with the dependent variables (Tables S3-S6 in Multimedia Appendix 1), as indicated by the standardized regression coefficient β, individuals with low educational levels, individuals with no spouse, individuals with ≥6 family members, farmers or fishermen, individuals with hypertension diagnosis, and occasional smokers did not exhibit low levels of drinking, indicating that the attenuation of the alcohol-suppressing effect of flushing in these categories was caused by the first factor. Conversely, the other categories showed relatively low drinking levels, suggesting that the attenuation in these categories may belong to the second factor.

Previous reports on university students or office workers in Korea and Japan have mentioned that the effect of restraining themselves from drinking alcohol weakens, even if they have ALDH2 deficiency, under pressure from their peers or seniors at drinking gatherings (East Asian drinking culture of forcing alcohol consumption); similarly, Irons et al [9] documented this phenomenon concerning parental alcohol consumption and misuse [1,10]. All these aspects are closely related to the categories in which attenuation occurred in this study. Our study is the first to confirm that the suppressing effect of flushing on alcohol consumption can be significantly attenuated for certain groups of people in a large population representing East Asians at a comprehensive country level. The study findings suggest that public health stakeholders in alcohol consumption behavior should identify such attenuation factors and vulnerable groups to seek out preventive practices and policies.

In this study, multinomial logistic regression allowed us to thoroughly examine the research phenomenon by providing a comprehensive set of pairwise comparison results with respect to the established reference categories. However, this model was more complex compared to the binary logistic regression model, making the interpretation of the analysis results more challenging. Furthermore, this investigation used data from the 2019 and 2020 KNHANES. The second-year survey (2020) coincided with the onset of the COVID-19 pandemic. While Korea did not implement a lockdown during this period, measures such as gathering bans led to a decrease in monthly drinking rates from 73.4% in 2019 to 70.2% in 2020. These public health policies may have imposed mental health burdens, potentially driving individuals toward substance use as coping mechanisms [27]. Despite the influence of the pandemic, our findings suggest that there were no remarkable differences between 2019 and 2020 in terms of key outcomes.

### Limitations

This study has several limitations. First, although ALDH2 deficiency prediction by the flushing questionnaire is known to be highly accurate overall and advantageous for large-scale group surveys in which it is difficult to apply genetic tests, the 40.11% or 44.42% of alcohol flushing rate in this study was somewhat higher than the 29% to 37% variant ALDH2 genotype rate of Koreans [28,29]. In the study conducted by Yokoyama et al [15] in 1997, people whose faces “sometimes” turn red after less drinking were also regarded as having ALDH2 deficiency, even if they never experienced flushing when they started drinking. However, even those with active ALDH2 may encounter flushing with minimal alcohol consumption depending on their current physiological state or owing to advancing age. Furthermore, a few individuals may also experience flushing due to the variant alcohol dehydrogenase enzyme. These factors may have slightly compromised the accuracy of the KNHANES flushing questionnaire in distinguishing inactive ALDH2 [6,30].

### Conclusions

Flushing, a prevalent genetic trait among East Asians, significantly inhibits alcohol consumption at a national level among Koreans, even after accounting for sociodemographic and health-related factors. However, this robust biological defense mechanism is limited or negligible in demographically vulnerable Koreans, potentially increasing their susceptibility to the group-1 carcinogen acetaldehyde. Health authorities should conduct targeted epidemiological studies to assess drinking patterns and disease profiles, particularly regarding alcohol-related cancers, and establish effective preventive measures tailored to this population. The generalizability of these findings to other East Asian communities should also be determined.

## Appendix

Multimedia Appendix 1 Additional tables presenting results from multinomial logistic regression analyses using data from the 2019-2020 Korean National Health and Nutrition Examination Survey.

## Abbreviations

ALDH2: aldehyde dehydrogenase 2
KNHANES: Korea National Health and Nutrition Examination Survey
OR: odds ratio

## Data Availability

The data sets analyzed during this study are available in the Korea National Health and Nutrition Examination Survey (KNHANES) repository.
