# Supplementary material for: Impact of Alcohol-Induced Facial Flushing Phenotype on Alcohol Consumption Among Korean Adults: 2-Year Cross-Sectional Study
Source: JMIR Public Health Surveill. 2024 Jul 31;10:e49826. doi: 10.2196/49826 (PMC11325126; doi:10.2196/49826)
Supplement: Multimedia Appendix 1 [file publichealth_v10i1e49826_app1.pdf]

Table S1. Changes in the impact of alcohol-induced facial flushing[a] on drinking frequency by year among Korean adults from 2019-2020 Korea National Health and Nutrition Examination Survey

Predictor variable	Statistics	Drinking frequency (Reference = did not drink in the past year)				
		<Once per month	Once per month	2–4 times per month	2–3 times per week	≥4 times per week
**Year*Flushing**	(*F* = 3.02; *df*=5,341; *P*=.011)					
2019	OR [95% CI]	1.07 [0.85,1.34]	0.78* [0.62,0.98]	0.56*** [0.47,0.68]	0.56*** [0.45,0.69]	0.50*** [0.39,0.64]
2020		0.88 [0.73,1.07]	0.60*** [0.47,0.76]	0.43*** [0.36,0.52]	0.31*** [0.25,0.39]	0.41*** [0.30,0.56]

[a] A genetic predisposition linked to aldehyde dehydrogenase 2 deficiency, manifesting as facial redness even with small amounts of alcohol. The identification of this phenotype relies on a two-step questionnaire outlined in the Method section.
Note. The sample for the Korea National Health and Nutrition Examination Survey (KNHANES) in 2019-2020 was selected through a complex sample design involving a multi-stage stratified cluster probability sampling method each year. The target population for the study was individuals aged ≥ 19 years and non-lifetime abstainers from alcohol consumption. Multinomial logistic regression analysis was conducted, incorporating complex sample design elements, including strata, clusters, and weights, to ensure unbiased results. The *F* value in parentheses represents the test statistic for Type 3 analysis of the interaction effect between the two variables under investigation.
* $P < .05$. ** $P < .01$. *** $P < .001$.

Table S2. Changes in the impact of alcohol-induced facial flushing[a] on drinking amount by year among Korean adults from 2019-2020 Korea National Health and Nutrition Examination Survey

Predictor variable	Statistics	Drinking amount (glasses per drinking occasion) (Reference = did not drink in the past year)				
		1 - 2	3 - 4	5 - 6	7 - 9	≥ 10
**Year*Flushing**	(*F* = 2.01; *df*=5,341; *P* =.077)					
2019	OR [95% CI]	1.04 [0.86,1.26]	0.73** [0.60,0.89]	0.63*** [0.50,0.79]	0.52*** [0.40,0.69]	0.36*** [0.28,0.47]
2020		0.77** [0.64,0.92]	0.51*** [0.41,0.62]	0.47*** [0.37,0.60]	0.36*** [0.28,0.45]	0.34*** [0.27,0.44]

[a] A genetic predisposition linked to aldehyde dehydrogenase 2 deficiency, manifesting as facial redness even with small amounts of alcohol. The identification of this phenotype relies on a two-step questionnaire outlined in the Method section.
Note. The sample for the Korea National Health and Nutrition Examination Survey (KNHANES) in 2019-2020 was selected through a complex sample design involving a multi-stage stratified cluster probability sampling method each year. The target population for the study was individuals aged ≥ 19 years and non-lifetime abstainers from alcohol consumption. Multinomial logistic regression analysis was conducted, incorporating complex sample design elements, including strata, clusters, and weights, to ensure unbiased results. The *F* value in parentheses represents the test statistic for Type 3 analysis of the interaction effect between the two variables under investigation.
* $P < .05$. ** $P < .01$. *** $P < .001$.

Table S3. Effects of other explanatory variables on drinking frequency among Korean adults from 2019 Korea National Health and Nutrition Examination Survey

Predictor variable	Category vs. reference category	Statistics	Drinking frequency (Reference = did not drink in the past year) <Once per month	Once per month	2 – 4 times per month	2 – 3 times per week	≥ 4 times per week
**Age**		β[a]	-10.55	−10.36	−14.22	−12.23	−5.58
		OR	0.97***	0.97***	0.96***	0.97***	0.99
		[95%CI]	[096,0.99]	[0.96,0.99]	[0.95,0.98]	[0.96,0.98]	[0.97,1.00]
**BMI**		β	2.27	-3.63*	-0.22	-2.17	-2.46
		OR	1.03	0.96*	1.00	0.98	0.97
		[95%CI]	[0.99,1.06]	[0.92,1.00]	[0.97,1.03]	[0.94,1.01]	[0.93,1.02]
**Sex**		β	1.67	6.68	9.01	9,72	17.77
		OR	1.15	1.77**	2.15***	2.29***	4.54***
		[95%CI]	[0.84,1.59]	[1.25,2.49]	[1.54,3.01]	[1.60,3.28]	[2.93,7.03]
**Spouse**	Living with a spouse vs. Never married	β	-2.36	−3.62	−0.74	3.20	5.80
		OR	0.81	0.72	0.94	1.34	1.69
		[95%CI]	[0.55,1.19]	[0.45,1.15]	[0.64,1.38]	[0.84,2.12]	[0.93,3.08]
	Living without a spo use vs. Never married	β	-0.13	−3.65	−1.24	-0.30	3.05
		OR	0.98	0.58*	0.83	0.96	1.57
		[95%CI]	[0.61,1.57]	[0.34,0.99]	[0.52,1.34]	[0.55,1.68]	[0.75,3.29]
**Educatio n**	Middle school vs. ≤Elementary school diploma	β	-2.11	−0.45	−0.79	1.18	−2.07
		OR	0.71	0.93	0.88	1.22	0.71
		[95%CI]	[0.47,1.05]	[0.54,1.60]	[0.55,1.39]	[0.75,1.97]	[0.44,1.15]
	High school vs. ≤Elementary school diploma	β	-3.57	−2.47	−3.48	−0.35	−10.92
		OR	0.73	0.80	0.73	0.97	0.38***
		[95%CI]	[0.52,1.02]	[0.52,1.24]	[0.48,1.13]	[0.63,1.49]	[0.23,0.61]
	>University vs. ≤Elementary school diploma	β	-4.74	−5.10	−4.60	−1.44	−16.25
		OR	0.67*	0.65	0.67	0.88	0.25***
		[95%CI]	[0.44,1.00]	[0.41,1.01]	[0.43,1.07]	[0.55,1.41]	[0.14,0.45]
**Occupati on**	Office worker vs. Administrator, profe ssional	β	-1.58	-0.21	2.46	1.72	3.32
		OR	0.82	0.97	1.36	1.24	1.52
		[95%CI]	[0.53,1.28]	[0.60,1.57]	[0.87,2.13]	[0.78,1.99]	[0.77,2.98]
	Service or sales work er vs. Administrator, profe ssional	β	0.44	-0.16	0.33	0.62	1.44
		OR	1.06	0.98	1.04	1.08	1.20
		[95%CI]	[0.66,1.70]	[0.56,1.71]	[0.66,1.66]	[0.65,1.79]	[0.60,2.38]
	Farmer, fisherman v s. Administrator, profe ssional	β	0.26	-0.78	-1.51	-0.80	1.39
		OR	1.08	0.79	0.63	0.78	1.53
		[95%CI]	[0.55,2.14]	[0.31,2.01]	[0.32,1.22]	[0.40,1.54]	[0.60,3.92]
	Mechanic, technician vs. Administrator, profe ssional	β	2.22	-0.30	0.02	0.98	-0.08
		OR	1.34	0.96	1.00	1.14	0.99
		[95%CI]	[0.82,2.18]	[0.57,1.61]	[0.61,1.66]	[0.67,1.93]	[0.50,1.96]
	Simple labor worker vs. Administrator, profe ssional	β	-1.20	-1.05	-0.78	0.21	-0.48
		OR	0.82	0.84	0.88	1.04	0.93
		[95%CI]	[0.48,1.41]	[0.47,1.52]	[0.50,1.56]	[0.58,1.84]	[0.43,1.98]
	Unemployed (house wife, student) vs. Administrator, profe ssional	β	-1.00	-2.35	-3.49	-4.73	-3.05
		OR	0.91	0.81	0.73	0.65	0.76
		[95%CI]	[0.60,1.39]	[0.52,1.24]	[0.49,1.08]	[0.42,1.02]	[0.38,1.49]
**Type of h ealth ins urance**	National health insur ance residence-based vs. Medical care	β	6.77	−1.25	−5.82	−1.00	2.49
		OR	1.92	0.89	0.57	0.91	1.27
		[95%CI]	[0.99,3.70]	[0.43,1.85]	[0.29,1.14]	[0.41,2.00]	[0.54,3.00]
	National health insur ance work-based vs. Medical care		8.34	0.90	−5.06	−1.84	−1.18
		β	2.18*	1.09	0.62	0.84	0.90
			[1.12,4.24]	[0.54,2.20]	[0.32,1.21]	[0.39,1.81]	[0.39,2.07]

		OR<br>[95%CI]					
**Smoking**	Daily vs. Never	β<br>OR<br>[95%CI]	-3.15<br>0.71<br>[0.48,1.05]	−0.49<br>0.95<br>[0.62,1.44]	5.79<br>1.89***<br>[1.35,2.66]	13.73<br>4.53***<br>[3.01,6.83]	18.60<br>7.75***<br>[4.72,12.71]
	Occasionally vs. Never	β<br>OR<br>[95%CI]	1.05<br>1.28<br>[0.50,3.26]	2.45<br>1.77<br>[0.67,4.68]	5.25<br>3.40**<br>[1.39,8.29]	7.85<br>6.24***<br>[2.42,16.13]	7.56<br>5.83**<br>[1.96,17.36]
	Smoked before but not presently vs. Never	β<br>OR<br>[95%CI]	-3.80<br>0.69*<br>[0.49,0.97]	0.47<br>1.05<br>[0.76,1.45]	3.15<br>1.36*<br>[1.00,1.85]	9.39<br>2.52***<br>[1.77,3.59]	13.50<br>3.78***<br>[2.37,6.03]
**Limitation on life activities due to health or disability**	Have vs. Do not have	β<br>OR<br>[95%CI]	-2.28<br>0.65*<br>[0.44,0.96]	-3.87<br>0.48*<br>[0.28,0.84]	-4.50<br>0.43***<br>[0.26,0.71]	-4.03<br>0.47**<br>[0.28,0.79]	-3.16<br>0.55<br>[0.29,1.03]
**Feeling stressed**	Very frequently vs. Seldomly	β<br>OR<br>[95%CI]	138<br>1.32<br>[0.75,2.30]	2.14<br>1.53<br>[0.82,2.86]	−0.94<br>0.83<br>[0.43,1.59]	1.26<br>1.29<br>[0.67,2.48]	4.30<br>2.35*<br>[1.05,5.27]
	Frequently vs. Seldomly	β<br>OR<br>[95%CI]	4.15<br>1.51*<br>[1.03,2.21]	4.61<br>1.58*<br>[1.04,2.39]	0.86<br>1.09<br>[0.77,1.55]	1.12<br>1.12<br>[0.73,1.72]	5.26<br>1.68*<br>[1.05,2.71]
	Occasionally vs. Seldomly	β<br>OR<br>[95%CI]	3.87<br>1.40*<br>[1.01,1.93]	6.26<br>1.71**<br>[1.20,2.46]	3.52<br>1.36*<br>[1.01,1.82]	2.96<br>1.29<br>[0.90,1.85]	0.91<br>1.08<br>[0.75,1.55]
**Number of major fatal illness[b]**	1 vs. Other including no response	β<br>OR<br>[95%CI]	-3.82<br>0.53***<br>[0.36,0.76]	-2.56<br>0.65<br>[0.42,1.01]	-5.24<br>0.42***<br>[0.29,0.60]	-3.56<br>0.55**<br>[0.35,0.86]	-3.73<br>0.54*<br>[0.30,0.96]
	≥2 vs. Other including no response	β<br>OR<br>[95%CI]	-1.03<br>0.58<br>[0.25,1.36]	-3.65<br>0.15*<br>[0.03,0.75]	-3.38<br>0.17*<br>[0.04,0.80]	-2.17<br>0.32<br>[0.10,1.04]	-2.15<br>0.32<br>[0.09,1.20]
**Hypertension diagnosis**	Present vs. Other including no response	β<br>OR<br>[95%CI]	-1.71<br>0.83<br>[0.62,1.11]	-0.43<br>0.95<br>[0.63,1.44]	2.21<br>1.27<br>[0.94,1.73]	3.03<br>1.39*<br>[1.01,1.92]	3.74<br>1.50*<br>[1.07,2.12]

[a] The statistic β represents a standardized regression coefficient, the absolute value of which reflects the degree of association between the predictor variable and the outcome variable (drinking frequency), which enhances direct comparability among multiple predictor variables in the multinomial logistic regression analysis. [b] The number of diagnoses by doctors for stomach, liver, colon, breast, cervical, lung, thyroid, and other cancers; stroke; myocardial infarction; or angina.

Note. The sample for the Korea National Health and Nutrition Examination Survey (KNHANES) in 2019-2020 was selected through a complex sample design involving a multi-stage stratified cluster probability sampling method each year. The target population for the study was individuals aged ≥ 19 years and non-lifetime abstainers from alcohol consumption in 2019. Multinomial logistic regression analysis was conducted, incorporating complex sample design elements, including strata, clusters, and weights, to ensure unbiased results. Pseudo-R^2 for the model was 0.30. All statistically significant confounding variables, as determined by Type 3 analysis of effects, are presented. There were no significant contrasts found in occupation, despite the overall test yielding significance in the Type 3 analysis of effects.

$^{*}P < .05$. $^{**}P < .01$. $^{***}P < .001$.

Table S4. Effects of other explanatory variables on drinking frequency among Korean adults from 2020 Korea National Health and Nutrition Examination Survey

Predictor variable	Category vs. reference category	Statistics	Drinking frequency (Reference = did not drink in the past year)				
			<Once per month	Once per month	2 – 4 times per month	2 – 3 times per week	≥ 4 times per week
**Age**		β[a]	-5.10	-18.43	-12.01	-15.53	-15.06
		OR	0.99*	0.95***	0.97***	0.96***	0.96***
		[95%CI]	[0.97,1.00]	[0.94,0.97]	[0.96,0.98]	[0.95,0.97]	[0.94,0.98]
**BMI**		β	3.30	2.33	-1.04	2.12	-4.07
		OR	1.04	1.03	0.99	1.02	0.96
		[95%CI]	[0.99,1.08]	[0.98,1.07]	[0.95,1.03]	[0.98,1.07]	[0.91,1.01]
**Sex**		β	-0.91	2.41	5.69	9.53	13.08
		OR	0.93	1.23	1.62***	2.24***	3.04***
		[95%CI]	[0.68,1.26]	[0.83,1.82]	[1.22,2.15]	[1.62,3.10]	[1.93,4.77]
**Spouse**	Living with a spouse vs. Never married	β	-2.65	4.34	-0.12	7.62	17.08
		OR	0.79	1.47	0.99	1.96**	4.51***
		[95%CI]	[0.54,1.16]	[0.96,2.25]	[0.67,1.46]	[1.25,3.06]	[2.40,8.48]
	Living without a spouse vs. Never married	β	0.88	5.44	2.30	2.88	9.58
		OR	1.14	2.20**	1.39	1.52	4.00***
		[95%CI]	[0.71,1.83]	[1.22,3.95]	[0.86,2.25]	[0.84,2.75]	[1.84,8.71]
**Education**	Middle school vs. ≤Elementary school diploma	β	-0.45	-1.30	-1.40	1.24	1.12
		OR	0.93	0.80	0.79	1.24	1.21
		[95%CI]	[0.61,1.41]	[0.45,1.43]	[0.47,1.32]	[0.74,2.06]	[0.63,2.33]
	High school vs. ≤Elementary school diploma	β	0.37	-3.96	-1.66	-1.34	-3.47
		OR	1.03	0.71	0.87	0.89	0.74
		[95%CI]	[0.69,1.55]	[0.42,1.18]	[0.55,1.35]	[0.58,1.38]	[0.38,1.44]
	>University vs. ≤Elementary school diploma	β	0.13	-4.46	-2.25	-0.37	-7.10
		OR	1.01	0.68	0.82	0.97	0.54
		[95%CI]	[0.66,1.55]	[0.38,1.22]	[0.55,1.24]	[0.61,1.54]	[0.27,1.11]
**Family size**	2 vs. 1	β	1.12	1.50	-1.34	-1.21	-7.46
		OR	1.12	1.16	0.88	0.89	0.48
		[95%CI]	[0.78,1.61]	[0.71,1.90]	[0.57,1.36]	[0.56,1.41]	[0.24,0.97]
	3 vs. 1	β	0.09	-1.63	-3.56	-4.84	-7.64
		OR	1.01	0.86	0.72	0.63	0.49*
		[95%CI]	[0.68,1.49]	[0.53,1.40]	[0.46,1.11]	[0.40,1.01]	[0.25,0.95]
	4 vs. 1	β	1.06	-3.25	-3.09	-5.45	-8.21
		OR	1.11	0.74	0.75	0.60*	0.46*
		[95%CI]	[0.70,1.74]	[0.44,1.21]	[0.47,1.20]	[0.37,0.97]	[0.23,0.93]
	5 vs. 1	β	-1.89	-3.57	-1.77	-1.78	-6.20
		OR	0.75	0.58	0.76	0.76	0.38*
		[95%CI]	[0.40,1.39]	[0.30,1.11]	[0.43,1.36]	[0.40,1.45]	[0.16,0.92]
	≥6 vs. 1	β	-2.21	-2.83	-2.87	-5.09	-2.21
		OR	0.53*	0.45	0.44	0.24**	0.53
		[95%CI]	[0.29,0.99]	[0.18,1.12]	[0.19,1.01]	[0.10,0.56]	[0.17,1.72]
**Occupation**	Office worker vs. Administrator, professional	β	1.54	3.71	2.10	3.17	2.22
		OR	1.22	1.62	1.32	1.51	1.34
		[95%CI]	[0.72,2.08]	[0.94,2.79]	[0.80,2.17]	[0.86,2.66]	[0.65,2.73]
	Service or sales worker vs. Administrator, professional	β	-0.78	-1.28	0.59	2.06	4.60
		OR	0.91	0.85	1.08	1.29	1.76
		[95%CI]	[0.59,1.40]	[0.51,1.43]	[0.68,1.70]	[0.80,2.07]	[0.89,3.49]
	Farmer, fisherman vs. Administrator, professional	β	-0.26	-2.75	-1.80	0.72	4.26
		OR	0.93	0.45	0.59	1.24	3.51*
		[95%CI]	[0.47,1.83]	[0.18,1.13]	[0.26,1.32]	[0.53,2.89]	[1.30,9.50]
	Mechanic, technician vs.	β	0.40	1.72	1.98	3.18	2.27
		OR	1.06	1.27	1.31	1.55	1.37

	Administrator, profe ssional	[95%CI]	[0.61,1.84]	[0.68,2.38]	[0.79,2.18]	[0.90,2.66]	[0.62,3.02]
	Simple labor worker vs. Administrator, profe ssional	*β*<br>OR<br>[95%CI]	-2.93<br>0.62<br>[0.36,1.05]	-0.74<br>0.88<br>[0.48,1.64]	-1.29<br>0.81<br>[0.48,1.36]	-0.29<br>0.95<br>[0.49,1.84]	-0.02<br>1.00<br>[0.41,2.41]
	Unemployed (house wife, student) vs. Administrator, profe ssional	*β*<br>OR<br>[95%CI]	-1.00<br>0.91<br>[0.64,1.30]	0.22<br>1.02<br>[0.67,1.55]	-0.53<br>0.95<br>[0.66,1.39]	-1.39<br>0.88<br>[0.58,1.34]	-0.88<br>0.92<br>[0.47,1.81]
**Househo ld incom e**	Low-Middle vs. Low	β<br>OR<br>[95%CI]	-2.25<br>0.76<br>[0.52,1.12]	1.40<br>1.19<br>[0.74,1.91]	0.44<br>1.06<br>[0.62,1.79]	0.50<br>1.06<br>[0.61,1.85]	-4.93<br>0.55<br>[0.27,1.13]
	Middle vs. Low	β<br>OR<br>[95%CI]	-0.58<br>0.94<br>[0.62,1.44]	3.61<br>1.45<br>[0.89,2.36]	1.34<br>1.15<br>[0.69,1.90]	4.39<br>1.57<br>[0.91,2.69]	-4.10<br>0.66<br>[0.33,1.30]
	Middle-High vs. Low	β<br>OR<br>[95%CI]	0.36<br>1.04<br>[0.69,1.55]	7.55<br>2.07***<br>[1.35,3.17]	4.95<br>1.61<br>[0.93,2.78]	5.20<br>1.65<br>[0.93,2.92]	2.76<br>1.30<br>[0.65,2.64]
	High vs. Low	β<br>OR<br>[95%CI]	2.90<br>1.31<br>[0.86,2.01]	10.05<br>2.56***<br>[1.61,4.07]	7.35<br>1.99*<br>[1.18,3.37]	8.98<br>2.32**<br>[1.30,4.12]	-0.17<br>0.98<br>[0.49,1.97]
**Number of houses owned**	1 vs. 0	β<br>OR<br>[95%CI]	3.24<br>1.32<br>[0.98,1.76]	5.77<br>1.63*<br>[1.12,2.38]	3.24<br>1.32<br>[0.95,1.83]	4.12<br>1.42<br>[0.99,2.03]	-1.00<br>0.92<br>[0.59,1.44]
	≥2 vs. 0	β<br>OR<br>[95%CI]	0.48<br>1.06<br>[0.71,1.59]	5.20<br>1.89*<br>[1.16,3.10]	1.91<br>1.26<br>[0.84,1.91]	1.46<br>1.20<br>[0.71,2.01]	1.78<br>1.24<br>[0.63,2.45]
**Type of health in surance**	National health insur ance residence-based vs. Medical care	*β*<br>OR<br>[95%CI]	6.89<br>1.90*<br>[1.00,3.59]	-3.90<br>0.70<br>[0.37,1.33]	-0.68<br>0.94<br>[0.52,1.71]	-1.81<br>0.85<br>[0.41,1.76]	11.28<br>2.85*<br>[1.14,7.12]
	National health insur ance work-based vs. Medical care	*β*<br>OR<br>[95%CI]	10.47<br>2.58**<br>[1.36,4.89]	1.98<br>1.20<br>[0.63,2.29]	3.89<br>1.42<br>[0.77,2.62]	2.04<br>1.20<br>[0.56,2.57]	13.93<br>3.53*<br>[1.33,9.35]
**Private health in surance**	Have vs. Do not have	β<br>OR<br>[95%CI]	1.08<br>1.15<br>[0.87,1.51]	2.14<br>1.31<br>[0.88,1.95]	5.02<br>1.89***<br>[1.35,2.64]	5.72<br>2.06***<br>[1.44,2.96]	1.48<br>1.21<br>[0.73,1.99]
**Smoking**	Daily vs. Never	*β*<br>OR<br>[95%CI]	1.67<br>1.20<br>[0.76,1.90]	7.10<br>2.21**<br>[1.34,3.64]	10.52<br>3.23***<br>[2.13,4.91]	15.56<br>5.68***<br>[3.83,8.41]	23.59<br>13.91***<br>[8.20,23.6 0]
	Occasionally vs. Never	*β*<br>OR<br>[95%CI]	6.11<br>4.37***<br>[2.05,9.30]	3.81<br>2.51<br>[0.76,8.25]	8.61<br>7.99***<br>[3.26,19.5 7	9.86<br>10.80***<br>[4.02,29.0 6]	9.79<br>10.62***<br>[3.33,33.9 1]
	Smoked before but n ot presently vs. Never	*β*<br>OR<br>[95%CI]	-2.45<br>0.79<br>[0.57,1.09]	1.53<br>1.16<br>[0.76,1.77]	4.03<br>1.48*<br>[1.05,2.08]	10.27<br>2.70***<br>[1.99,3.67]	11.59<br>3.07***<br>[1.95,4.84]
**Weight control effort**	To lose weight vs. Never tried	*β*<br>OR<br>[95%CI]	0.70<br>1.06<br>[0.79,1.44]	-1.18<br>0.90<br>[0.67,1.23]	4.03<br>1.41*<br>[1.05,1.90]	1.04<br>1.09<br>[0.80,1.49]	1.91<br>1.18<br>[0.76,1.82]
	To maintain weight vs. tried	*β*<br>OR<br>[95%CI]	-0.10<br>0.99<br>[0.73,1.34]	0.85<br>1.09<br>[0.77,1.54]	1.14<br>1.12<br>[0.83,1.53]	1.91<br>1.22<br>[0.88,1.69]	0.25<br>1.03<br>[0.64,1.66]
	To gain weight vs. Never tried	*β*<br>OR<br>[95%CI]	-0.57<br>0.89<br>[0.50,1.59]	-3.34<br>0.50<br>[0.24,1.04]	-2.54<br>0.59<br>[0.32,1.07]	-3.14<br>0.52<br>[0.27,1.01]	-1.78<br>0.69<br>[0.33,1.44]
**Limitatio n on life activities due to h ealth or**	Have vs. Do not have	*β*<br>OR<br>[95%CI]	-1.45<br>0.75<br>[0.50,1.14]	-4.47<br>0.42**<br>[0.22,0.81]	-2.18<br>0.65<br>[0.39,1.09]	-4.69<br>0.40**<br>[0.23,0.70]	-2.77<br>0.58<br>[0.28,1.21]

disability

**Hypertension diagnosis**	Present vs. Other including no response	β<br>OR<br>[95%CI]	-0.45<br>0.95<br>[0.74,1.23]	-1.29<br>0.87<br>[0.59,1.29]	1.43<br>1.17<br>[0.88,1.55]	2.12<br>1.26<br>[0.89,1.78]	5.49<br>1.81**<br>[1.22,2.68]

[a] The statistic β represents a standardized regression coefficient, the absolute value of which reflects the degree of association between the predictor variable and the outcome variable (drinking frequency), which enhances direct comparability among multiple predictor variables in the multinomial logistic regression analysis.

Note. The sample for the Korea National Health and Nutrition Examination Survey (KNHANES) in 2019-2020 was selected through a complex sample design involving a multi-stage stratified cluster probability sampling method each year. The target population for the study was individuals aged ≥ 19 years and non-lifetime abstainers from alcohol consumption in 2020. Multinomial logistic regression analysis was conducted, incorporating complex sample design elements, including strata, clusters, and weights, to ensure unbiased results. Pseudo-R^2 for the model was 0.33. All statistically significant confounding variables, as determined by Type 3 analysis of effects, are presented. There were no significant contrasts found in BMI and education, despite the overall tests indicating significance in the Type 3 analysis of effects.

$^{*}P < .05$. $^{**}P < .01$. $^{***}P < .001$.

Table S5. Effects of other explanatory variables on drinking amount among Korean adults from 2019 Korea National Health and Nutrition Examination Survey

Predictor variable	Category vs. reference category	Statistics	Drinking amount (glasses per drinking occasion) (reference = did not drink in the past year)				
			1 – 2	3 – 4	5 – 6	7 - 9	≥ 10
**Age**		β^a<br>OR<br>[95%CI]	-3.44<br>0.99<br>[0.98,1.00]	−12.94<br>0.97***<br>[0.95,0.98]	−13.38<br>0.97***<br>[0.95,0.98]	−18.16<br>0.95***<br>[0.94,0.97]	−32.48<br>0.92***<br>[0.90,0.93]
**BMI**		β^a<br>OR<br>[95%CI]	-1.97<br>0.98<br>[0.95,1.01]	-1.21<br>0.99<br>[0.96,1.02]	-0.74<br>0.99<br>[0.95,1.03]	-1.85<br>0.98<br>[0.94,1.02]	3.98<br>1.05*<br>[1.01,1.09]
**Off-day sleep hours**		β^a<br>OR<br>[95%CI]	-1.04<br>0.97<br>[0.92,1.03]	-3.82<br>0.91**<br>[0.85,0.97]	-1.39<br>0.97<br>[0.90,1.04]	-3.33<br>0.92<br>[0.84,1.00]	-3.43<br>0.92<br>[0.83,1.01]
**Sex**		β<br>OR<br>[95%CI]	-0.79<br>0.94<br>[0.70,1.26]	8.96<br>2.14***<br>[1.52,3.02]	12.23<br>2.83***<br>[1.99,4.04]	17.92<br>4.60***<br>[3.04,6.96]	17.85<br>4.57***<br>[3.02,6.92]
**Education**	Middle school vs. ≤Elementary school diploma	β<br>OR<br>[95%CI]	-1.86<br>0.74<br>[0.52,1.03]	−0.93<br>0.86<br>[0.54,1.38]	0.00<br>1.00<br>[0.56,1.77]	1.25<br>1.23<br>[0.74,2.06]	2.41<br>1.49<br>[0.71,3.13]
	High school vs. ≤Elementary school diploma	β<br>OR<br>[95%CI]	-3.95<br>0.70*<br>[0.52,0.96]	−4.73<br>0.66<br>[0.42,1.02]	−1.75<br>0.86<br>[0.52,1.40]	−3.19<br>0.75<br>[0.49,1.16]	2.04<br>1.20<br>[0.61,2.35]
	>University vs. ≤Elementary school diploma	β<br>OR<br>[95%CI]	-3.13<br>0.77<br>[0.54,1.08]	−8.73<br>0.47**<br>[0.29,0.78]	−7.05<br>0.55*<br>[0.31,0.95]	−3.44<br>0.75<br>[0.48,1.16]	−0.84<br>0.93<br>[0.46,1.88]
**Occupation**	Office worker vs. Administrator, professional	β<br>OR<br>[95%CI]	-1.31<br>0.85<br>[0.56,1.29]	0.38<br>1.05<br>[0.64,1.72]	2.97<br>1.45<br>[0.86,2.44]	3.18<br>1.49<br>[0.94,2.38]	4.59<br>1.78*<br>[1.07,2.97]
	Service or sales worker vs. Administrator, professional	β<br>OR<br>[95%CI]	-0.84<br>0.90<br>[0.56,1.44]	−0.93<br>0.89<br>[0.54,1.46]	0.53<br>1.07<br>[0.62,1.84]	3.07<br>1.47<br>[0.84,2.56]	3.28<br>1.50<br>[0.85,2.66]
	Farmer, fisherman vs. Administrator, professional	β<br>OR<br>[95%CI]	-0.41<br>0.88<br>[0.49,1.57]	−1.52<br>0.63<br>[0.33,1.19]	0.05<br>1.02<br>[0.37,2.79]	1.20<br>1.45<br>[0.55,3.82]	0.95<br>1.34<br>[0.50,3.58]
	Mechanic, technician vs. Administrator, professional	β<br>OR<br>[95%CI]	-0.80<br>0.90<br>[0.54,1.49]	−0.01<br>1.00<br>[0.61,1.62]	0.60<br>1.08<br>[0.61,1.93]	4.20<br>1.73*<br>[1.04,2.88]	0.70<br>1.10<br>[0.59,2.02]

	Simple labor worker vs.<br>Administrator, professional	β<br>OR<br>[95%CI]	-1.37<br>0.80<br>[0.48,1.33]	−2.23<br>0.69<br>[0.39,1.23]	−0.20<br>0.97<br>[0.50,1.86]	2.18<br>1.43<br>[0.71,2.89]	−0.80<br>0.88<br>[0.41,1.87]
	Unemployed (house wife, student) vs.<br>Administrator, professional	β<br>OR<br>[95%CI]	-1.95<br>0.84<br>[0.56,1.26]	-5.43<br>0.61*<br>[0.42,0.89]	−2.75<br>0.78<br>[0.49,1.24]	−2.55<br>0.79<br>[0.49,1.27]	−4.22<br>0.68<br>[0.42,1.11]
**Private health insurance**	Have vs. Do not have	β<br>OR<br>[95%CI]	-0.35<br>0.96<br>[0.72,1.27]	0.04<br>1.01<br>[0.73,1.39]	3.36<br>1.51<br>[1.00,2.29]	4.17<br>1.67*<br>[1.08,2.58]	5.20<br>1.90*<br>[1.15,3.13]
**Smoking**	Daily vs. Never	β<br>OR<br>[95%CI]	-5.68<br>0.54***<br>[0.38,0.76]	4.57<br>1.65*<br>[1.12,2.45]	8.11<br>2.44***<br>[1.62,3.68]	14.20<br>4.77***<br>[3.15,7.24]	20.54<br>9.59***<br>[6.17,14.89]
	Occasionally vs. Never	β<br>OR<br>[95%CI]	0.23<br>1.05<br>[0.41,2.70]	4.78<br>3.05*<br>[1.16,8.00]	5.44<br>3.55*<br>[1.30,9.69]	6.64<br>4.70**<br>[1.62,13.65]	11.06<br>13.18***<br>[4.85,35.78]
	Smoked before but not presently vs. Never	β<br>OR<br>[95%CI]	-2.06<br>0.82<br>[0.59,1.13]	2.32<br>1.26<br>[0.91,1.75]	4.55<br>1.57*<br>[1.11,2.20]	10.46<br>2.80***<br>[1.97,3.99]	15.26<br>4.49***<br>[2.92,6.92]
**Weight control effort**	To lose weight vs. Never tried	β<br>OR<br>[95%CI]	0.78<br>1.07<br>[0.84,1.37]	1.67<br>1.15<br>[0.87,1.53]	2.06<br>1.19<br>[0.88,1.62]	3.58<br>1.36<br>[0.99,1.87]	3.23<br>1.32<br>[0.95,1.84]
	To maintain weight vs.<br>Tried	β<br>OR<br>[95%CI]	1.85<br>1.22<br>[0.94,1.58]	2.26<br>1.27<br>[0.93,1.73]	0.73<br>1.08<br>[0.75,1.57]	-0.30<br>0.97<br>[0.66,1.41]	-1.13<br>0.89<br>[0.59,1.34]
	To gain weight vs. Never tried	β<br>OR<br>[95%CI]	-1.17<br>0.81<br>[0.53,1.24]	-5.24<br>0.39***<br>[0.24,0.64]	-1.39<br>0.78<br>[0.43,1.40]	-4.37<br>0.45*<br>[0.23,0.89]	-2.93<br>0.59<br>[0.31,1.12]
**Limitation on life activities due to health or disability**	Have vs. Do not have	β<br>OR<br>[95%CI]	-2.35<br>0.64*<br>[0.46,0.91]	−3.00<br>0.57*<br>[0.35,0.92]	−5.53<br>0.35**<br>[0.19,0.66]	−5.69<br>0.34**<br>[0.17,0.69]	−4.16<br>0.46*<br>[0.22,0.93]
**Suicide attempt**	Yes vs. No	β<br>OR<br>[95%CI]	1.30<br>2.45<br>[0.54,11.07]	-16.06[b]<br>0.00***<br>[0.00,0.00]	0.10<br>1.07<br>[0.10,11.83]	0.06<br>1.04<br>[0.12,9.43]	0.06<br>1.04<br>[0.12,9.43]
**Number of major fatal illness[c]**	1 vs. Other, including no response	β<br>OR<br>[95%CI]	-3.03<br>0.6**<br>[0.44,0.82]	-4.61<br>0.46***<br>[0.31,0.69]	-4.62<br>0.46**<br>[0.28,0.77]	-4.74<br>0.45**<br>[0.26,0.78]	-3.06<br>0.6<br>[0.32,1.11]
	≥2 vs. Other, including no response	β<br>OR<br>[95%CI]	-1.68<br>0.42*<br>[0.18,0.95]	-2.95<br>0.21*<br>[0.06,0.74]	-2.75<br>0.24<br>[0.05,1.07]	-4.44<br>0.1*<br>[0.01,0.85]	-0.08<br>0.96<br>[0.15,6.33]
**Hypertension diagnosis**	Present vs. Other including no response	β<br>OR<br>[95%CI]	-1.31<br>0.87<br>[0.68,1.11]	1.59<br>1.19<br>[0.86,1.64]	3.06<br>1.39<br>[0.98,1.98]	2.95<br>1.38<br>[0.95,2.01]	5.41<br>1.80*<br>[1.14,2.85]

[a] The statistic β represents a standardized regression coefficient, the absolute value of which reflects the degree of association between the predictor variable and the outcome variable (drinking amount), which enhances direct comparability among multiple predictor variables in the multinomial logistic regression analysis. [b] There were no cases of suicide attempts within the past year among individuals in the '3-4 glasses' drinking category, indicating that the estimated statistics may not be reliable. [c] The number of diagnoses by doctors for stomach, liver, colon, breast, cervical, lung, thyroid, and other cancers; stroke; myocardial infarction; or angina.
Note. The sample for the Korea National Health and Nutrition Examination Survey (KNHANES) in 2019-2020 was selected through a complex sample design involving a multi-stage stratified cluster probability sampling method each year. The target population for the study was individuals aged ≥ 19 years and non-lifetime abstainers from alcohol consumption in 2019. Multinomial logistic regression analysis was conducted, incorporating complex sample design elements, including strata, clusters, and weights, to ensure unbiased results. Pseudo-R^2 for the model was 0.40. The categories of missing data were omitted. All statistically significant confounding variables, as determined by Type 3 analysis of effects, are presented.
* $P < .05$. ** $P < .01$. *** $P < .001$.

Table S6. Effects of other explanatory variables on drinking amount among Korean adults from 2020 Korea National Health and Nutrition Examination Survey

Predictor variable	Category vs. reference category	Statistics	Drinking amount (glasses per drinking occasion) (reference = did not drink in the past year)				
			1 – 2	1 – 2	1 – 2	1 – 2	1 – 2
**Age**		β[a]	-6.51	-8.24	-14.86	-18.28	-29.22
		OR	0.98**	0.98**	0.96***	0.95***	0.93***
		[95%CI]	[0.97,1.00]	[0.97,0.99]	[0.95,0.98]	[0.94,0.97]	[0.91,0.94]
**BMI**		β	0.14	0.63	-0.68	1.32	6.15
		OR	1.00	1.01	0.99	1.02	1.07*
		[95%CI]	[0.97,1.04]	[0.96,1.06]	[0.95,1.04]	[0.97,1.06]	[1.01,1.13]
**Sex**		β	-1.75	2.85	9.91	11.23	17.17
		OR	0.86	1.27	2.32***	2.59***	4.29***
		[95%CI]	[0.64,1.15]	[0.93,1.75]	[1.59,3.38]	[1.81,3.71]	[2.91,6.34]
**Education**	Middle school vs. ≤Elementary school diploma	β	-0.05	-1.84	2.90	-1.85	1.50
		OR	0.99	0.73	1.64	0.73	1.29
		[95%CI]	[0.66,1.48]	[0.43,1.25]	[0.84,3.23]	[0.41,1.29]	[0.59,2.85]
	High school vs. ≤Elementary school diploma	β	-1.13	-3.89	7.05	-3.18	-3.02
		OR	0.91	0.71	1.85*	0.76	0.77
		[95%CI]	[0.64,1.29]	[0.47,1.09]	[1.01,3.41]	[0.42,1.36]	[0.36,1.65]
	>University vs. ≤Elementary school diploma	β	-0.77	-2.53	4.65	-4.76	-2.60
		OR	0.94	0.81	1.49	0.67	0.80
		[95%CI]	[0.66,1.33]	[0.53,1.22]	[0.81,2.76]	[0.36,1.24]	[0.36,1.79]
**Occupation**	Office worker vs. Administrator, professional	β	2.38	2.91	-0.22	2.25	4.28
		OR	1.36	1.46	0.97	1.34	1.75
		[95%CI]	[0.84,2.21]	[0.85,2.52]	[0.53,1.79]	[0.72,2.51]	[0.93,3.27]
	Service or sales worker vs. Administrator, professional	β	0.03	0.11	-1.36	-0.33	4.02
		OR	1.00	1.01	0.85	0.96	1.64
		[95%CI]	[0.66,1.54]	[0.62,1.66]	[0.50,1.43]	[0.59,1.57]	[0.95,2.84]
	Farmer, fisherman vs. Administrator, professional	β	0.08	-0.37	-1.82	1.52	0.97
		OR	1.03	0.90	0.58	1.56	1.33
		[95%CI]	[0.56,1.87]	[0.39,2.07]	[0.22,1.57]	[0.65,3.76]	[0.49,3.61]
	Mechanic, technician vs. Administrator, professional	β	-0.94	1.52	1.80	3.28	5.34
		OR	0.88	1.23	1.28	1.57	2.09*
		[95%CI]	[0.50,1.53]	[0.74,2.05]	[0.70,2.33]	[0.90,2.75]	[1.09,3.99]
	Simple labor worker vs. Administrator, professional	β	-1.23	-0.88	-4.44	-2.13	-2.11
		OR	0.82	0.87	0.48*	0.70	0.71
		[95%CI]	[0.50,1.35]	[0.46,1.62]	[0.25,0.93]	[0.38,1.30]	[0.34,1.45]
	Unemployed (housewife, student) vs.	β	-0.35	-0.54	-3.79	-1.38	-0.37
		OR	0.97	0.95	0.71	0.88	0.97
		[95%CI]	[0.69,1.37]	[0.64,1.43]	[0.43,1.18]	[0.56,1.41]	[0.59,1.58]

	Administrator, professional						
**Household income**	Low-Middle vs. Low	β<br>OR<br>[95%CI]	1.74<br>1.23<br>[0.87,1.74]	-3.38<br>0.67<br>[0.39,1.12]	-4.57<br>0.58<br>[0.31,1.08]	-4.03<br>0.61<br>[0.34,1.10]	-1.79<br>0.81<br>[0.38,1.69]
	Middle vs. Low	β<br>OR<br>[95%CI]	3.06<br>1.37<br>[0.94,2.00]	-1.5<br>0.86<br>[0.53,1.38]	-0.7<br>0.93<br>[0.49,1.76]	-3.07<br>0.73<br>[0.42,1.29]	1.83<br>1.21<br>[0.61,2.39]
	Middle-High vs. Low	β<br>OR<br>[95%CI]	4.57<br>1.55*<br>[1.09,2.21]	0.68<br>1.07<br>[0.64,1.80]	3.11<br>1.35<br>[0.70,2.62]	1.01<br>1.1<br>[0.60,2.02]	5.84<br>1.76<br>[0.85,3.64]
	High vs. Low	β<br>OR<br>[95%CI]	7.11<br>1.95***<br>[1.33,2.86]	3.13<br>1.34<br>[0.79,2.28]	4.97<br>1.59<br>[0.83,3.06]	1.78<br>1.18<br>[0.64,2.19]	8.39<br>2.19*<br>[1.07,4.52]
**Type of health insurance**	National health insurance residence-based vs. Medical care	*β*<br>OR<br>[95%CI]	1.27<br>1.13<br>[0.65,1.95]	4.73<br>1.55<br>[0.73,3.29]	2.46<br>1.26<br>[0.56,2.82]	6.59<br>1.85<br>[0.87,3.93]	-1.43<br>0.88<br>[0.39,1.98]
	National health insurance work-based vs. Medical care	*β*<br>OR<br>[95%CI]	5.1<br>1.59<br>[0.91,2.78]	8.37<br>2.13<br>[0.98,4.62]	7.76<br>2.02<br>[0.91,4.46]	12.24<br>3.03**<br>[1.42,6.47]	1.59<br>1.16<br>[0.50,2.67]
**Private health insurance**	Have vs. Do not have	β<br>OR<br>[95%CI]	1.11<br>1.15<br>[0.86,1.55]	3.92<br>1.64**<br>[1.16,2.32]	4.86<br>1.85**<br>[1.23,2.78]	4.42<br>1.75**<br>[1.20,2.55]	4.96<br>1.87**<br>[1.16,3.01]
**Smoking**	Daily vs. Never	*β*<br>OR<br>[95%CI]	-0.54<br>0.94<br>[0.60,1.49]	6.79<br>2.13**<br>[1.33,3.43]	14.76<br>5.19***<br>[3.27,8.23]	19.49<br>8.80***<br>[5.40,14.34]	24.31<br>15.07***<br>[9.65,23.53]
	Occasionally vs. Never	*β*<br>OR<br>[95%CI]	3.36<br>2.25<br>[0.92,5.51]	7.53<br>6.16***<br>[2.80,13.53]	10.42<br>12.38***<br>[4.67,32.82]	10.72<br>13.29***<br>[4.84,36.52]	12.77<br>21.83***<br>[8.16,58.39]
	Smoked before but not presently vs. Never	*β*<br>OR<br>[95%CI]	-1.69<br>0.85<br>[0.63,1.15]	4.03<br>1.48*<br>[1.03,2.12]	6.37<br>1.85**<br>[1.26,2.72]	12.66<br>3.41***<br>[2.34,4.96]	11.64<br>3.09***<br>[2.12,4.49]
**Limitation on life activities due to health or disability**	Have vs. Do not have	*β*<br>OR<br>[95%CI]	-2.61<br>0.60*<br>[0.40,0.90]	-4.85<br>0.39***<br>[0.22,0.68]	-1.43<br>0.76<br>[0.37,1.55]	-1.97<br>0.68<br>[0.37,1.24]	-1.18<br>0.80<br>[0.42,1.51]
**Number of major fatal illness[b]**	1 vs. Other including no response	*β*<br>OR<br>[95%CI]	-0.57<br>0.91<br>[0.63,1.31]	-2.71<br>0.63*<br>[0.40,0.99]	-1.86<br>0.73<br>[0.43,1.23]	-5.89<br>0.37***<br>[0.21,0.64]	-4.30<br>0.48*<br>[0.24,0.98]
	≥2 vs. Other including no response	*β*<br>OR<br>[95%CI]	-0.73<br>0.64<br>[0.25,1.65]	-1.27<br>0.46<br>[0.12,1.71]	-2.33<br>0.24<br>[0.03,1.98]	-0.79<br>0.62<br>[0.14,2.82]	-1.07<br>0.52<br>[0.07,3.73]
**Hypertension diagnosis**	Present vs. Other including no response	*β*<br>OR<br>[95%CI]	-1.08<br>0.89<br>[0.69,1.16]	2.28<br>1.28<br>[0.94,1.74]	0.40<br>1.04<br>[0.71,1.53]	5.10<br>1.73**<br>[1.23,2.43]	4.30<br>1.59*<br>[1.06,2.39]
**Dyslipidemia diagnosis**	Present vs. Other, including no response	β<br>OR<br>[95%CI]	-0.24<br>0.97<br>[0.75,1.27]	-5.26<br>0.56***<br>[0.40,0.78]	-0.59<br>0.94<br>[0.62,1.43]	-1.48<br>0.85<br>[0.59,1.22]	-0.76<br>0.92<br>[0.58,1.45]

[a] The statistic β represents a standardized regression coefficient, the absolute value of which reflects the degree of association between the predictor variable and the outcome variable (drinking amount), which enhances direct comparability among multiple predictor variables in the multinomial logistic regression analysis. [b] The number of diagnoses by doctors for stomach, liver, colon, breast, cervical, lung, thyroid, and other cancers; stroke; myocardial infarction; or angina.
Note. The sample for the Korea National Health and Nutrition Examination Survey (KNHANES) in 2019-2020 was selected through a complex sample design involving a multi-stage stratified cluster probability sampling method each year. The target population for the study was individuals aged ≥ 19 years and non-lifetime abstainers from alcohol consumption in 2020. Multinomial logistic regression analysis was conducted, incorporating complex sample design elements, including strata, clusters, and weights, to ensure unbiased results. Pseudo-R^2 for the model was 0.40. All statistically significant confounding variables, as determined by Type 3 analysis of effects, are presented.
$^{*}P < .05$. $^{**}P < .01$. $^{***}P < .001$.

Table S7. Changes in the impact of alcohol-induced facial flushing[a] on drinking frequency at different levels of demographic and health-related variables among Korean adults from 2020 Korea National Health and Nutrition Examination Survey (simple effects analysis)

Predictor variable	Statistics	Drinking frequency (Reference = did not drink in the past year)				
		<Once per month	Once per month	2–4 times per month	2–3 times per week	≥4 times per week
**Sex*Flushing**	(F = 3.95; df=5,341; P=.002)					
Male	OR [95% CI]	1.18 [0.83,1.68]	0.53** [0.35,0.80]	0.40*** [0.29,0.54]	0.25*** [0.18,0.35]	0.32*** [0.22,0.46]
Female		0.76* [0.59,0.97]	0.64** [0.46,0.87]	0.40*** [0.30,0.52]	0.34*** [0.24,0.49]	0.50* [0.27,0.92]
**Education*Flushing**	(F = 3.57; df=20,326; P<.001)					
≤Elementary school diploma	OR [95% CI]	0.61 [0.37,1.01]	0.71 [0.34,1.47]	0.62 [0.35,1.08]	0.65 [0.35,1.21]	0.47 [0.20,1.13]
Middle school diploma		0.76 [0.38,1.50]	0.28** [0.12,0.65]	1.02 [0.53,1.98]	0.51 [0.25,1.04]	0.50 [0.20,1.25]
High school diploma		0.75 [0.52,1.10]	0.67 [0.44,1.01]	0.42*** [0.30,0.60]	0.41*** [0.28,0.60]	0.45** [0.27,0.76]
≥University diploma		1.06 [0.79,1.42]	0.51*** [0.35,0.74]	0.36*** [0.27,0.49]	0.18*** [0.13,0.26]	0.30*** [0.16,0.55]
**Family size* Flushing**	(F = 1.54; df=25,321; P=.049)					
1	OR [95% CI]	1.02 [0.56,1.87]	0.49 [0.23,1.04]	0.48** [0.29,0.79]	0.32*** [0.17,0.58]	0.80 [0.40,1.60]
2		1.10 [0.76,1.57]	0.73 [0.49,1.10]	0.67* [0.48,0.93]	0.44*** [0.30,0.66]	0.46** [0.27,0.77]
3		0.78 [0.54,1.12]	0.45** [0.27,0.73]	0.36*** [0.25,0.53]	0.33*** [0.22,0.50]	0.33*** [0.17,0.62]
4		1.02 [0.71,1.45]	0.89 [0.56,1.41]	0.41*** [0.28,0.61]	0.32*** [0.20,0.52]	0.36** [0.18,0.72]
5		0.42* [0.18,0.94]	0.32* [0.13,0.76]	0.22*** [0.09,0.53]	0.08*** [0.03,0.25]	0.11** [0.03,0.42]
≥6		0.56 [0.20,1.53]	0.69 [0.14,3.35]	0.47 [0.11,2.07]	0.26 [0.06,1.22]	1.19 [0.31,4.63]
**Occupation* Flushing**	(F = 1.59; df=35,311; P=.022)					
Administrator, professional	OR [95% CI]	0.79 [0.47,1.33]	0.32*** [0.17,0.62]	0.28*** [0.15,0.51]	0.19*** [0.10,0.35]	0.17*** [0.06,0.49]
Office worker		1.30 [0.65,2.61]	0.75 [0.37,1.51]	0.39** [0.21,0.73]	0.18*** [0.08,0.39]	0.57 [0.22,1.52]
Service or sales worker		0.80 [0.44,1.45]	0.64 [0.32,1.29]	0.59 [0.34,1.02]	0.31*** [0.16,0.60]	0.30** [0.13,0.69]

Farmer, fishermen		3.01* [1.03,8.84]	0.37 [0.09,1.47]	0.98 [0.22,4.42]	0.34 [0.11,1.07]	0.38 [0.11,1.38]
Mechanic, technician		1.53 [0.69,3.36]	0.50 [0.22,1.17]	0.38** [0.19,0.76]	0.27*** [0.13,0.57]	0.29** [0.12,0.70]
Simple labor worker		0.72 [0.37,1.43]	0.64 [0.29,1.40]	0.41** [0.21,0.79]	0.40* [0.18,0.87]	0.68 [0.25,1.90]
Unemployed (housewife, student)		0.71* [0.52,0.97]	0.62* [0.42,0.91]	0.42*** [0.30,0.59]	0.43*** [0.30,0.64]	0.53 [0.28,1.01]
**Household income* Flushing**	(*F* = 2.22; *df*=20,326; *P*=.002)					
Low	OR [95% CI]	0.54* [0.32,0.89]	0.65 [0.33,1.29]	0.52* [0.28,0.96]	0.74 [0.40,1.36]	0.58 [0.30,1.14]
Low-Middle		0.93 [0.60,1.44]	0.46* [0.24,0.89]	0.65 [0.41,1.04]	0.41** [0.24,0.70]	0.28** [0.12,0.67]
Middle		0.94 [0.63,1.39]	0.90 [0.56,1.47]	0.61* [0.41,0.92]	0.28*** [0.17,0.46]	0.53 [0.25,1.12]
Middle-High		1.05 [0.70,1.59]	0.66 [0.42,1.04]	0.40*** [0.26,0.62]	0.31*** [0.20,0.50]	0.56 [0.30,1.04]
High		0.76 [0.52,1.13]	0.40*** [0.24,0.67]	0.25*** [0.17,0.37]	0.22*** [0.14,0.33]	0.20*** [0.11,0.39]
**Smoking*Flushing**	(*F* = 2.59; *df*=15,331; *P*=.001)					
Daily	OR [95% CI]	0.61 [0.29,1.30]	0.26*** [0.12,0.55]	0.19*** [0.10,0.38]	0.10*** [0.05,0.19]	0.15*** [0.07,0.32]
Occasionally		1.77 [0.22,14.48]	0.17 [0.01,2.50]	0.65 [0.12,3.54]	0.85 [0.14,5.15]	0.09 [0.01,1.25]
Smoked before but not presently		1.38 [0.87,2.18]	0.59 [0.32,1.06]	0.48*** [0.33,0.70]	0.26*** [0.17,0.40]	0.31*** [0.18,0.54]
Never		0.84 [0.66,1.06]	0.66** [0.49,0.89]	0.38*** [0.29,0.50]	0.34*** [0.24,0.48]	0.49* [0.26,0.94]

[a] A genetic predisposition linked to aldehyde dehydrogenase 2 deficiency, manifesting as facial redness even with small amounts of alcohol. The identification of this phenotype relies on a two-step questionnaire outlined in the Method section.

Note. The sample for the Korea National Health and Nutrition Examination Survey (KNHANES) in 2019-2020 was selected through a complex sample design involving a multi-stage stratified cluster probability sampling method each year. The target population for the study was individuals aged ≥ 19 years and non-lifetime abstainers from alcohol consumption in 2020. Multinomial logistic regression analysis was conducted, followed by simple-effect analysis for variables showing a significant interaction effect with flushing. To ensure unbiased results, complex sample design elements, including strata, clusters, and weights, were incorporated in the multinomial logistic regression analysis. The *F* value in parentheses represents the test statistic for Type 3 analysis of the interaction effect between the two variables under investigation.

* $P < .05$. ** $P < .01$. *** $P < .001$.

Table S8. Changes in the impact of alcohol-induced facial flushing[a] on drinking amount at different levels of demographic and health-related variables among Korean adults from 2020 Korea National Health and Nutrition Examination Survey (simple effects analysis)

Predictor variable	Statistics	Drinking amount (glasses per drinking occasion) (Reference = did not drink in the past year)				
		1–2	3–4	5–6	7–9	≥10
**Age[b]*Flushing[a]**	(*F* = 4.92; *df*=5,341; *P*<.001)					
**Education*Flushing**	(*F* = 1.75; *df*=20,326; *P*=.025)					
≤Elementary school diploma	OR [95% CI]	0.57* [0.35,0.94]	0.60 [0.33,1.08]	0.31* [0.11,0.84]	0.54 [0.28,1.02]	2.47 [0.98,6.18]
Middle school diploma		0.63 [0.37,1.08]	0.70 [0.31,1.63]	1.08 [0.42,2.80]	0.36** [0.16,0.77]	0.56 [0.24,1.29]
High school diploma		0.78 [0.54,1.14]	0.56** [0.38,0.82]	0.49*** [0.33,0.75]	0.44*** [0.30,0.65]	0.32*** [0.21,0.48]
≥University diploma		0.76* [0.59,0.99]	0.40*** [0.28,0.56]	0.40*** [0.28,0.59]	0.25*** [0.17,0.38]	0.28*** [0.19,0.42]

**Private health insurance*Flushing**	(F = 2.25; df=5,341; P=.049)					
Have	OR<br>[95% CI]	0.70***<br>[0.57,0.85]	0.45***<br>[0.36,0.56]	0.40***<br>[0.31,0.53]	0.31***<br>[0.24,0.40]	0.32***<br>[0.25,0.42]
Does not have		0.92<br>[0.61,1.40]	0.72<br>[0.43,1.20]	1.15<br>[0.58,2.30]	0.67<br>[0.37,1.22]	0.38**<br>[0.20,0.74]

[a] A genetic predisposition linked to aldehyde dehydrogenase 2 deficiency, manifesting as facial redness even with small amounts of alcohol. The identification of this phenotype relies on a two-step questionnaire outlined in the Method section. [b] Although age had a significant interaction with flushing, a simple effect analysis could not be performed because age was a continuous variable. Instead, the interaction effect between flushing and age could be visually observed by comparing the difference in expected probability between the flushing group and the non-flushing group over age at each level of drinking frequency (Figure 1).

Note. The sample for the Korea National Health and Nutrition Examination Survey (KNHANES) in 2019-2020 was selected through a complex sample design involving a multi-stage stratified cluster probability sampling method each year. The target population for the study was individuals aged ≥ 19 years and non-lifetime abstainers from alcohol consumption in 2020. Multinomial logistic regression analysis was conducted, followed by simple-effect analysis for variables showing a significant interaction effect with flushing. To ensure unbiased results, complex sample design elements, including strata, clusters, and weights, were incorporated in the multinomial logistic regression analysis. The F value in parentheses represents the test statistic for Type 3 analysis of the interaction effect between the two variables under investigation. A significant interaction effect was observed for the variable 'Number of deadly diseases,' but omitted due to unstable results caused by zero or near-zero observations in certain categories reflecting the rarity in the population.

$^{*}P < .05$. $^{**}P < .01$. $^{***}P < .001$.

Table S9. Effect of alcohol-induced facial flushing[a] on drinking frequency among Korean adults by gender from 2019 Korea National Health and Nutrition Examination Survey

Predictor variable	Category vs. reference category	Statistics	Drinking frequency (Reference = did not drink in the past year)				
			<Once per month	Once per month	2 – 4 times per month	2 – 3 times per week	≥ 4 times per week
**Alcohol-induced facial flushing (Male)**	Presence vs. Absence of simple model	β[b]<br>OR<br>[95%CI]	1.01<br>1.13<br>[0.77,1.64]	-4.13<br>0.61**<br>[0.43,0.87]	−6.85<br>0.45***<br>[0.33,0.60]	−7.99<br>0.39***<br>[0.28,0.53]	−8.83<br>0.35***<br>[0.25,0.50]
	Presence vs. Absence of complex model	β<br>OR<br>[95%CI]	2.00<br>1.27<br>[0.84,1.91]	−3.86<br>0.63*<br>[0.44,0.91]	−6.85<br>0.45***<br>[0.32,0.63]	−8.82<br>0.35***<br>[0.25,0.50]	−10.11<br>0.30***<br>[0.21,0.45]
**Alcohol-induced facial flushing (Female)**	Presence vs. Absence of simple model	β<br>OR<br>[95%CI]	0.34<br>1.04<br>[0.81,1.35]	−1.30<br>0.85<br>[0.64,1.14]	−4.32<br>0.58***<br>[0.45,0.76]	−2.85<br>0.70*<br>[0.53,0.93]	−3.26<br>0.67<br>[0.37,1.20]
	Presence vs. Absence of complex model	β<br>OR<br>[95%CI]	0.42<br>1.05<br>[0.81,1.38]	−1.12<br>0.87<br>[0.64,1.19]	−4.57<br>0.57***<br>[0.43,0.76]	−3.36<br>0.66**<br>[0.48,0.90]	−5.22<br>0.52*<br>[0.28,0.99]

[a] A genetic predisposition linked to aldehyde dehydrogenase 2 deficiency, manifesting as facial redness even with small amounts of alcohol. The identification of this phenotype relies on a two-step questionnaire outlined in the Method section. [b] The statistic β represents a standardized regression coefficient, the absolute value of which reflects the degree of association between the predictor variable and the outcome variable (drinking frequency), which enhances direct comparability among multiple predictor variables in the multinomial logistic regression analysis.

Note. The sample for the Korea National Health and Nutrition Examination Survey (KNHANES) in 2019-2020 was selected through a complex sample design involving a multi-stage stratified cluster probability sampling method each year. The target population for the study was individuals aged ≥ 19 years and non-lifetime abstainers from alcohol consumption in 2019. Multinomial logistic regression analysis was conducted based on gender (gender-stratified analysis). To ensure unbiased results, complex sample design elements, including strata, clusters, and weights, were incorporated in the multinomial logistic regression analysis.

$^{*}P < .05$. $^{**}P < .01$. $^{***}P < .001$.
